# *ACAA1* knockout increases the survival rate of KPC mice by activating autophagy

**DOI:** 10.1016/j.molmet.2025.102237

**Published:** 2025-08-21

**Authors:** Ho Lee, Mingyu Kang, Sung Hoon Sim, Joon Hee Kang, Wonyoung Choi, Jung Won Chun, Woosol Hong, Chaeyoung Kim, Woojin Ham, Jeong Hwan Park, Eun-Byeol Koh, Yoon Jeon, Sang Myung Woo, Soo-Youl Kim

**Affiliations:** 1 Division of Cancer Biology, Research Institute, National Cancer Center, Goyang, Republic of Korea; 2 Department of Cancer Biomedical Science, Graduate School of Cancer Science and Policy, National Cancer Center, Goyang, Republic of Korea; 3 New Cancer Cure-Bio Co., Goyang, Republic of Korea; 4 Division of Rare and Refractory Cancer, Research Institute, National Cancer Center, Goyang, Republic of Korea; 5 Division of Clinical Research, Research Institute, National Cancer Center, Goyang, Republic of Korea; 6 Laboratory Animal Research Facility, Research Institute, National Cancer Center, Goyang, Republic of Korea; 7 Center for Liver and Pancreatobiliary Cancer, National Cancer Center, Goyang, Republic of Korea

**Keywords:** ACAA1, Peroxisome, Fatty acid oxidation, Pancreatic cancer, Autophagy

## Abstract

**Objectives:**

We found that the levels of the peroxisomal fatty acid oxidation (FAO) marker in pancreatic ductal adenocarcinoma (PDAC) patients were higher than those in healthy individuals, based on tissue microarray analysis. This study investigates FAO in preclinical in vitro and in vivo models.

**Methods:**

To examine the role of FAO in the peroxisome, we created acetyl-coenzyme A acyltransferase (ACAA1) knockout mice, crossed them with KPC mice, and monitored their survival rates. Additionally, we tested a mouse xenograft model with ACAA1 knockdown in human PDAC cells.

**Results:**

In normal cells, *ACAA1* knockdown did not affect oxygen consumption. In contrast, in PDAC cells, *ACAA1* knockdown reduced the oxygen consumption rate by up to 60% and decreased ATP production by up to 70%. This suggests that peroxisomes in PDAC supply various acyl-carnitines for FAO in mitochondria. In PDAC cells, *ACAA1* knockdown lowered ATP levels, resulting in mTOR inactivation and autophagy induction. Additionally, *ACAA1* knockdown significantly increased LC3-II levels, leading to growth retardation in mouse xenograft models. *Acaa1a*^*+/−*^ mice showed a median survival increase of 3 weeks after crossing *Acaa1a*^*+/−*^ with KPC mice (*Kras*^G12D/+^; *Trp53*^R172H/+;^*Pdx1*-Cre, a genetically engineered mice model for PDAC).

**Conclusions:**

*ACAA1* knockdown inhibited tumor growth by triggering autophagy, which supported the survival of KPC mice. The most important benefit of targeting ACAA1 is that it blocks tumor growth specifically in cancer cells without harming normal cell energy metabolism.

## Highlights

• Levels of peroxisomal FAO markers in PDAC patients were higher than in normals.
• *ACAA1* knockdown reduced ATP levels, leading to mTOR inactivation and induction of autophagy.
• *ACAA1* knockdown in PDAC cells significantly increased LC3-II, resulting in growth retardation in mice xenograft models.
• *Acaa1*^+/−^ mice increased 3 weeks of median survival rate by crossing *Acaa1*^+/−^ with KPC mice.
• In summary, *ACAA1* knockdown suppressed tumor growth by activating autophagy, which promoted the survival of KPC mice.

## 1 Introduction

There are conflicting reports about the role of autophagy in tumors, with some suggesting that autophagy suppresses tumor growth, while others propose it promotes tumor growth. However, these two claims are flawed because they are based on different contexts. One context involves tumor development due to the loss of autophagy, while the other concerns tumor regrowth caused by autophagy activation through chemotherapy. First, many early studies showed that normal cells could become tumorous because of the buildup of cellular damage when autophagy factors are deficient, especially in the absence of stressors that induce autophagy. The initial idea that autophagy suppresses tumors came from the discovery of frequent loss-of-function mutations in BECN1 in breast cancer cell lines [1]. Additionally, the loss of Atg7 resulted in tumor formation due to the accumulation of cellular damage in the liver [2]. These findings suggest that autophagy acts as a direct factor in preventing tumor formation but is not necessarily involved in causing cell death in response to chemotherapy. Second, autophagy alone is not enough for cancer cells to regrow; it has to be activated along with the growth switch that depends on biosynthesis using building blocks from autophagy [3,4]. Initially, autophagy induction slows tumor growth through autophagolysis; however, over time, the cancer can regrow by utilizing the various building blocks and energy sources provided by autophagy [5]. Therefore, cancer cells need strong signaling and a lot of ATPs to regrow during anticancer drug treatment.

Previously, we demonstrated that cancer cells depend on fatty acid oxidation (FAO) for ATP production instead of glycolysis [[6], [7], [8]]. Inhibition of FAO decreased ATP production in cancer cells in a dose-dependent way, while ATP levels stayed the same without glucose, even though lactate levels dropped by over 80% [7]. We found that inhibiting mitochondrial FAO through CAC (carnitine-acylcarnitine carrier; CACT, carnitine-acylcarnitine translocase; gene name *SLC25A20*) knockdown caused an accumulation of mid-chain and long-chain acyl-carnitines in pancreatic ductal adenocarcinoma (PDAC) cells, resulting in a substantial decrease in ATP production, which in turn inactivated mTOR signaling. The short-chain and mid-chain acyl-carnitines are generated from very long-chain or long-chain fatty acids via peroxisome FAO [9]. The acylated carnitine with a mid-chain fatty acid in the peroxisome can be used as a substrate for β-oxidation in the mitochondria through transport via CAC [10]. In this study, to assess whether peroxisomal FAO is essential for cancer survival, *ACAA1* was knocked down, as it is involved in the last step of β-oxidation that specifically targets peroxisomal FAO. We also examined whether *ACAA1* knockdown significantly triggers autophagy due to reduced ATP production from FAO inhibition and whether *Acaa1a*^*+/−*^ mice improve the overall survival of KPC mice through crossbreeding.

## 2 Materials and methods

### 2.1 Cell culture and transfection

Human pancreatic cancer cell lines MIA PaCa-2 (CRL-1420), SW1990 (CRL-2172), SU.86.86 (CRL-1837), AsPC-1 (CRL-1682), BxPC-3 (CRL-1687), Capan-1 (HTB-79), Capan-2 (HTB-80), HPAC (CRL-2119) and the hTERT-HPNE (representing normal human pancreatic epithelium; CRL-4023) cell line were obtained from the American Type Culture Collection (ATCC, Manassas, VA, USA). The hTERT-HPNE cell line (HPNE) is an hTERT-immortalized epithelial cell derived from the pancreas of a 52-year-old male patient [11]. HPNE cells use glucose for oxidative phosphorylation (OxPhos) as their primary energy source, like normal cells. Nestin is a specific marker for HPNE cells. The Nestin protein, a marker of neuronal stem cells, was found to be expressed by rare cells in the ducts and islets [12], and cultures of Nestin-positive cells derived from the pancreas can self-organize into clusters containing insulin and glucagon [12,13]. Although they are not entirely normal primary cells due to immortalisation, they are not cancer cells and possess normal metabolic properties, so we used these cells as normal cells for comparison with cancer cells.

PA-TU-8998S(ACC 204) and PA-TU-8988T(ACC 162) were obtained from DSMZ (Braunschweig, GER). A549 (Non-Small Cell Lung Carcinoma, CCL-185), PC-3 (Prostate Cancer, CRL-1435), and HCT-8 (Colorectal Cancer, CCL-244) were obtained from ATCC (Manassas, VA, USA). The human hepatocellular carcinoma cell SNU-449(00449) was obtained from the Korean Cell Line Bank (Seoul, KOR). Lenti-X™ 293T cells were obtained from the TaKaRa (Tokyo, Japan). MIA PaCa-2, SW1990, and Lenti-X™ 293T cells were grown in high glucose DMEM (SH30243.01; Hyclone, Logan, UT, USA) containing 10% fetal bovine serum (FBS; SH30070.03, HyClone), penicillin, and streptomycin. hTERT-HPNE (CRL-4023) was grown in 75% glucose-free DMEM (D-5030, Sigma–Aldrich, St. Louis, MO, USA) supplemented with 2 mM l-glutamine and 1.5 g/L sodium bicarbonate, plus 25% Medium M3 Base (Incell Corp. Texas, USA) containing 5% fetal bovine serum, 5.5 mM d-glucose (G8270, Sigma–Aldrich), and human recombinant EGF (E9644, Sigma–Aldrich). Except for the cell lines mentioned above, all other cell lines were cultured in RPMI-1640 medium supplemented with 10% fetal bovine serum (FBS) and 1% penicillin-streptomycin. All cells were maintained at 37 °C in a humidified atmosphere containing 5% CO_2_.

Lentiviral transduction was used to generate pancreatic cancer cell lines with stable knockdown of the *ACAA1* gene. Lentiviral *ACAA1* shRNA plasmids (TRCN0000036072 and TRCN0000036073) were purchased from Sigma–Aldrich (St. Louis, MO, USA). Lenti-X™ 293T cells were cultured and transfected with psPAX2, pMD2.G, and pLKO.1-puro-*ACAA1* shRNA lentiviral plasmids using using lipofectamine 3000 reagent (Thermo Fisher Scientific, Waltham, MA, USA) according to the manufacturer's instructions. 48 h after transfection, the culture media containing lentiviral particles were collected and centrifuged at 1500 g for 30 min. The supernatant was filtered using a 0.45 μm filter and then concentrated using PEG-it Virus Precipitation Solution (LV825A-1, System Biosciences, Palo Alto, USA) following the manufacturer's protocol. Pancreatic cancer cells were infected with the concentrated *ACAA1* shRNA lentivirus particles, and 48 h after infection, the transfected cells were selected with 1 μg/ml puromycin. The knockdown efficiency was confirmed by western blotting. siRNA duplexes targeting human *ACAA1* were transfected into cells at a final concentration of 40 nM for 48 h using Lipofectamine 3000 reagent according to the manufacturer's instructions. The *ACAA1* siRNA sequence is as follows (5′-3′): GUUUCCAAGCUGAGAUUGU. The negative siRNA sequence is as follows (5′3′): CCUCGUGCCGUUCCAUCAGGUAG.

### 2.2 Animal studies

#### 2.2.1 Generation of *Acaa1a*^+/−^ and *Acaa1a*^+/−^;KPC mice

Generation of *Acaa1a* knockout mice: A mixture of Cas9 protein (100 ng/μl) and sgRNAs (50 ng/μl each) was introduced into mouse zygotes via zygotic electroporation [14]. The sequences of the four sgRNAs used are as follows: #1: 5′-TAG TAT GCA GGA CAC TAC TT-3′, #2: 5′-AGC TGA ACC CTA GTA TGC AG-3′, #3: 5′-GGG TGT GTT ACC CGG CCC AG-3′, #4: 5′-TGC CTC TGG GCC GGG TAA CA-3'. Cas9 protein (EnGen Cas9 NLS) was purchased from New England Biolabs (NEB, Ipswich, MA, USA). sgRNAs were synthesized using a T7 in vitro transcription kit (NEB, Ipswich, MA, USA). Exon deletions in F1 mice were detected through TA cloning followed by sequencing. The pancreatic cancer mouse model, *Kras*^*G12D*^; *Trp53*^*R172H*^; and *Pdx1-Cre* (KPC), was used to examine the role of Acaa1a in the development and progression of pancreatic cancer. The KPC mouse model has been previously reported [15], but a brief explanation will be provided here. *Kras*^*G12D*^ mice (B6.129-Kras^tm4Tyj^/Nci) and *Trp53*^*R172H*^ mice (124S4-Trp53^tm2Tyj^/Nci) were obtained from the NCI Mouse Repository. Pdx1-Cre mice (B6.FVB-Tg(Pdx1-Cre)6Tuv/J, #014647) were obtained from The Jackson Laboratory. *Kras*^*G12D*^*; Pdx1-Cre* (KC) mice were generated by crossing *Kras*^*G12D*^ mice with *Pdx1-Cre* mice. KPC mice were generated by crossing KC mice with *Trp53*^*R172H*^ mice.

To generate *Acaa1a* heterozygous KC mice, KC mice were crossed with *Acaa1a*^*+/−*^ mice. Subsequently, *Trp53*^*R172H*^ mice were crossed with *Acaa1a*^*+/−*^ mice to produce *Trp53*^*R172H*^*; Acaa1a*^*+/−*^ mice, which were then crossed with *Acaa1a*^*+/−*^; KC mice to generate *Acaa1a*^*−/−*^; KPC mice. Initially, we designed an experiment to examine the anticancer efficacy by crossing KPC with *Acaa1a*^*+/+*^ or *Acaa1a*^*−/−*^ mice. However, since we confirmed that the expression of the ACAA1 protein was significantly reduced by more than 50% in *Acaa1a*^*+/−*^ mice, we determined that it would be sufficient to compare the anticancer efficacy by crossing KPC with either *Acaa1a*^*+/+*^ or *Acaa1a*^*+/−*^. The study was reviewed and approved by the Institutional Animal Care and Use Committee (IACUC) of the National Cancer Center Research Institute (protocols: NCC-22-588C-004), an AAALAC International-accredited facility that adheres to the Institute of Laboratory Animal Resources guidelines [16].

#### 2.2.2 Phenotype of *Acaa1a*^+/+^ and *Acaa1a*^+/−^

The *Acaa1a* mutant allele involves a deletion of exon 3, which causes a premature translational stop codon. This mutation produces a truncated Acaa1a protein consisting of only the N-terminal 88 amino acids of the full-length 424 amino acid wild-type protein. However, Western blot analysis did not detect the truncated protein. These findings support the interpretation that our model is functionally null regarding the mutant allele. Consistent with prior data from the International Mouse Phenotyping Consortium (IMPC, mousephenotype.org), *Acaa1a*^*+/−*^ mice do not show any obvious phenotype under normal conditions, and *Acaa1a*^*−/−*^ mice have also been reported to lack notable phenotypes, reinforcing the idea that loss of *Acaa1a* is well tolerated in healthy tissues.

#### 2.2.3 Establishment of PDAC xenograft mouse model

The antitumor efficacy of *ACAA1* gene knockdown or pharmacological inhibition was assessed using a xenograft model established with the human pancreatic cancer cell line MIA PaCa-2. Balb/c-nu/nu mice (Orient, Seoul, Korea, aged 6–8 weeks) were used for the mouse xenograft study using human PDAC cells. This study was reviewed and approved by the Institutional Animal Care and Use Committee of the National Cancer Center Research Institute (protocols: NCC-24-1014, NCC-24-1058-001). Briefly, MIA PaCa-2 (1 × 10^7^) was resuspended in 100 μL of PBS. Matrigel (BD Biosciences, Franklin Lakes, NJ, USA) was diluted with cold PBS to a final concentration of 50%, and a 1:1 mixture of the cell suspension and the diluted Matrigel was prepared on ice. Next, 200 μL of this mixture was injected subcutaneously into mice using a 1 ml syringe. MIA PaCa-2 cell lines with stable knockdown of the *ACAA1* gene were established as detailed in the Methods section and used to evaluate the anti-cancer efficacy of *ACAA1* knockdown. Mice were injected with pLKO.1-puro_scramble shRNA cells and pLKO.1-puro_*ACAA1* shRNA#1 or #2 cells. Tumor size was measured starting one week after tumor implantation. To evaluate the antitumor efficacy of the inhibitor, MIA PaCa-2 cells were inoculated into mice, and two weeks after implantation, the animals were randomized into control and Trimetazidine-treated groups (n = 7 per group). The control group received a vehicle (100% distilled water). In contrast, the treatment groups received Trimetazidine at doses of 25 or 50 mg/kg via oral gavage once daily, five days per week, for four weeks. Primary tumor size was measured weekly using calipers, and tumor volume (V) was calculated using the formula: V = (A × B^2^)/2, where V represents tumor volume (mm^3^), A is the longest diameter, and B is the shortest diameter."

### 2.3 Immunohistochemistry

Immunohistochemistry (IHC) was performed on formalin-fixed paraffin-embedded (FFPE) pancreas tissues from mice and tissue microarray (TMA) sections. A pancreatic cancer tissue array (PA2072a) was purchased from Tissue Array (Derwood, MD, US). Briefly, the sections were deparaffinized in xylene, dehydrated in ethanol, and rehydrated with water. Antigen retrieval was performed by immersing the slides in antigen retrieval buffer (10 mM sodium citrate, 0.05% Tween 20, pH 6.0) at 95 °C for 5 min. Endogenous peroxidases were blocked with 0.03% hydrogen peroxide, and nonspecific binding was blocked with 2% bovine serum albumin (BSA) in Tris-buffered saline with 0.1% Triton X-100 (TBST, pH 7.6) for 30 min. The sections were incubated with the primary antibody for 1.5 h at room temperature. After washing, the sections were incubated with the 3,3-diaminobenzidine chromogen (DAB) system to develop the stain for 5–20 min. The sections were counterstained with Mayer's hematoxylin for 30–60 s. Finally, the sections were dehydrated through 95% ethanol, followed by 100% ethanol, cleared in xylene, and mounted. TMAs are exempt from review by the National Cancer Center Institutional Review Board (IRB) under the following conditions: TMAs are used for research purposes, and samples are provided anonymously or unidentifiable. The TMAs we analyzed were provided anonymously by a US supplier and are therefore exempt from IRB review. The primary antibodies used in this experiment, their sources, and dilution ratios were as follows: ACAA1 (PA5-29956, Thermo Fisher Scientific, Waltham, MA, USA, 1:200), ACOX1 (10957-1-AP, Proteintech, 1:200), Ki-67 (ab15580, Abcam, Cambridge, UK, 1:1000), and LC3B (18725-1-AP, Proteintech, 1:750). The stained human pancreatic cancer tissue microarray (TMA) and tissue sections from the mouse model were scanned at high resolution using the Vectra Polaris multispectral imaging system (Akoya Biosciences, Marlborough, MA, US) and the Motic Easy Scan digital slide scanner (Motic, Kowloon Bay, Hong Kong). Protein expression was evaluated based on the pathological assessment of tissue morphology and staining patterns in pancreatic cancer tissues. The inForm Image Analysis Software (Akoya Biosciences, Marlborough, MA, US) was used to quantify the IHC results. H-scores were determined by considering the percentage of positively stained cells and staining intensity. The significance of CPT1A expression across different groups was tested with one-way ANOVA in GraphPad Prism version 10.3.1.

### 2.4 Oxygen consumption rate analysis

An Oxygen Consumption Rate (OCR) analysis was conducted following the established protocol [15]. 1–2 × 10^4^ cells were seeded in each well of a Seahorse microplate (103794-100, Agilent Technologies, California, USA) and incubated for 48 h. For determination of oxygen consumption rate (OCR), cells were incubated in XF base DMEM (103575-100, Agilent Technologies, California, USA) or RPMI (103576-100, Agilent Technologies, California, USA) medium supplemented with 10 mM glucose, 1 mM sodium pyruvate, and 2 mM l-glutamine. Then, cells were equilibrated in a non-CO_2_ incubator for 1 h before starting the assay. During the incubation, the Seahorse XF Cell Mito Stress Test Kit (103015-100, Agilent Technologies, California, USA), along with the mitochondrial inhibitors oligomycin (1 μM), FCCP (0.5–1 μM), and rotenone/antimycin A (0.5 μM) dissolved in XF base medium, was injected into the XFe96 sensor cartridge. Finally, normalization was performed with the SRB assay. To evaluate the effect of increased intracellular FAO caused by higher fatty acid uptake on ATP production, 200 mM behenic acid (216941, Sigma–Aldrich, St. Louis, MO, USA) and stearic acid (S4751, Sigma–Aldrich) were prepared by dissolving in 100% ethanol at 70 °C for 30 min. The stock solutions were then diluted 1:10 into serum-free DMEM containing 10% fatty acid-free BSA (A0281, Sigma–Aldrich) to create 20 mM behenic acid–BSA or stearic acid–BSA complex solutions. Before cell treatment, the FFA–BSA complexes were warmed in a 37 °C water bath for 30 min.

### 2.5 Mitochondrial membrane potential activity (Δψm)

Mitochondrial membrane potential was measured using tetramethylrhodamine-ethyl ester (TMRE, 87917, Sigma–Aldrich, St. Louis, US) staining. The assay method was performed as previously published [15]. Control and *ACAA1* knockdown cells were seeded in a 4-well chambered cover glass (155382, Thermo Fisher Scientific, Waltham, MA, US) with 0.5 ml of culture media. TMRE (40 nM) and Hoechst 33342 (5 μg/ml) were added to the culture medium and incubated for 15 min at 37 °C in a CO_2_ incubator. For the negative control, 50 μM trifluoromethoxy carbonylcyanide phenylhydrazone (FCCP, C2920, Sigma–Aldrich, St. Louis, MO, USA) was added to serum-free media for 15 at 37 °C in a CO_2_ incubator before TMRE staining. Live cell imaging was performed using the LSM780 Laser Scanning Microscope and Axio Observer Z1 (Carl Zeiss, Oberkochen, Germany). The relative intensity of TMRE was normalized to the arithmetic mean intensity (from ZEN software 3.4). The experiments were performed in triplicate, and statistical significance between control and experimental groups was assessed using one-way ANOVA in GraphPad Prism version 10.3.1.

### 2.6 Immunoblotting

Immunoblotting was conducted using the established protocol [7]. The primary antibodies used in this experiment, their sources, and dilution ratios were as follows: Total OXPHOS cocktail (ab110411, Abcam, Cambridge, UK 1:1000), β-actin (sc-47778, Santa Cruz Biotechnology, Dallas, TX, USA, 1:1000), ACAA1 (PA5-29956, Thermo Fisher Scientific, Waltham, MA, USA, 1:1000), mTOR (2972S, Cell Signaling Technology, Massachusetts, USA, 1:1000), Phospho mTOR (Ser2448) (2971S, Cell Signaling Technology, 1:1000), and GAPDH (A300-639A, Bethyl Laboratories, Montgomery, TX, USA, 1:1000). After primary antibody incubation, the membrane was washed three times with TBST and then incubated with HRP-conjugated secondary antibodies for 1 h at room temperature. Five additional washes with TBST followed. Chemiluminescent detection was performed using Dyne ECL Chemi (GBE C200 or GBE D50, Dyne Bio, Seongnam, Korea). Images were captured using the FUSION Solo 4 WL imaging system (Vilber Lourmat, France).

### 2.7 Colony formation assay

A Colony Formation Assay was conducted following the established protocol [17,18]. Cells were seeded in 6-well plates at a density of 0.5–1 × 10^3^ cells per well and cultured for two weeks without changing the medium (2 ml per well). After incubation, cells were gently washed twice with phosphate-buffered saline (PBS) and fixed with methanol for 10 min. Colonies were then stained with 0.1% crystal violet solution for 5 min, followed by PBS washes. Once the plates were completely air-dried, the stained colonies were eluted using an elution buffer composed of 50% ethanol, 40% distilled water, and 10% acetic acid for quantification. 100 μl of the eluate was transferred to a 96-well plate, and the absorbance was measured at 595 nm using a microplate reader. Each experiment was performed in triplicate, and the growth of pancreatic cancer cell lines under different conditions was analyzed using one-way ANOVA in GraphPad Prism version 10.3.1.

### 2.8 Sulforhodamine B (SRB) assay

Cells (1 × 10^4^ in 100 μL) were seeded into 96-well microplates. After 24 h, the drugs were prepared at the appropriate concentrations, and 100 μL of each solution was added to each well. The plates were then incubated in a CO_2_ incubator. Next, the cells were fixed by adding 50 μL of cold 50% (w/v) trichloroacetic acid (final concentration 10%) and incubated for at least 1 h at 4 °C. The microplates were washed five times with distilled water. Then, 100 μL of sulforhodamine B solution in 1% acetic acid was added to each well and allowed to stain for 10 min at room temperature. Following staining, the wells were washed five times with 1% acetic acid and thoroughly dried. Finally, the stained cells were solubilized with a 10 mM Trizma base, and the absorbance was measured at 515 nm using a microplate reader.

### 2.9 Immunofluorescence

Cells were grown on coverslips and transfected with *ACAA1* siRNA for 48 h. For rescue experiments, acetyl-CoA was added during the last 24 h of the transfection period. The cells were fixed with 4% paraformaldehyde for 15 min at room temperature, followed by staining with an anti-LC3B specific antibody (3868S, Cell Signaling Technology, Massachusetts, USA, 1:1000). Alexa Fluor™ ® 488 conjugated secondary antibody (Thermo Fisher Scientific, USA) was used for visualization. Nuclei were counterstained with Hoechst 33342 (blue). Confocal imaging was performed using the LSM780 Laser Scanning Microscope and Axio Observer Z1 (Carl Zeiss, Oberkochen, Germany). LC3-II puncta per cell were quantified using the ImageJ software.

### 2.10 Quantitation of energy metabolism and acyl-CoA-related metabolites using liquid chromatography-tandem mass spectrometry (LC-MS/MS)

Metabolites related to energy metabolism and Acetyl-CoA were analyzed using an LC-MS/MS system equipped with the 1290 HPLC system (Agilent, Waldbronn, Germany) and QTRAP 5500 (AB Sciex, Toronto, Canada), along with a reverse-phase column (Synergi Fusion RP 50 × 2 mm). Mobile phases A and B consisted of 5 mM ammonium acetate in water and 5 mM ammonium acetate in methanol, respectively. The separation gradient was as follows: hold at 0% B for 5 min, increase from 0% to 90% B over 2 min, hold at 90% B for 8 min, decrease from 90% to 0% B over 1 min, then hold at 0% B for 9 min. The LC flow rate was 70 μL/min, increasing to 140 μL/min between 7 and 15 min; the column temperature was maintained at 23 °C. Multiple reaction monitoring (MRM) was performed in negative ion mode, and the extracted ion chromatogram (EIC) corresponding to each metabolite's specific transition was used for quantification. The area under the curve for each EIC was normalized to that of the EIC of the internal standard. The peak area ratio of each metabolite to the internal standard was further normalized to the protein amount. Data analysis was conducted using Analyst 1.7.1 software.

### 2.11 Statistical analysis

All statistical analyses were performed using GraphPad Prism 10 software (GraphPad Software Inc., San Diego, CA, USA). Survival rates were calculated using the Kaplan–Meier method and compared using the log-rank test. Differences were assessed by one-way analysis of variance (ANOVA). P values ∗< 0.05, ∗∗< 0.01, ∗∗∗< 0.001, and ∗∗∗∗< 0.0001 were considered statistically significant.

## 3 Results

### 3.1 The peroxisome FAO was active in pancreatic cancer compared to normal tissue

**Figure 1:**
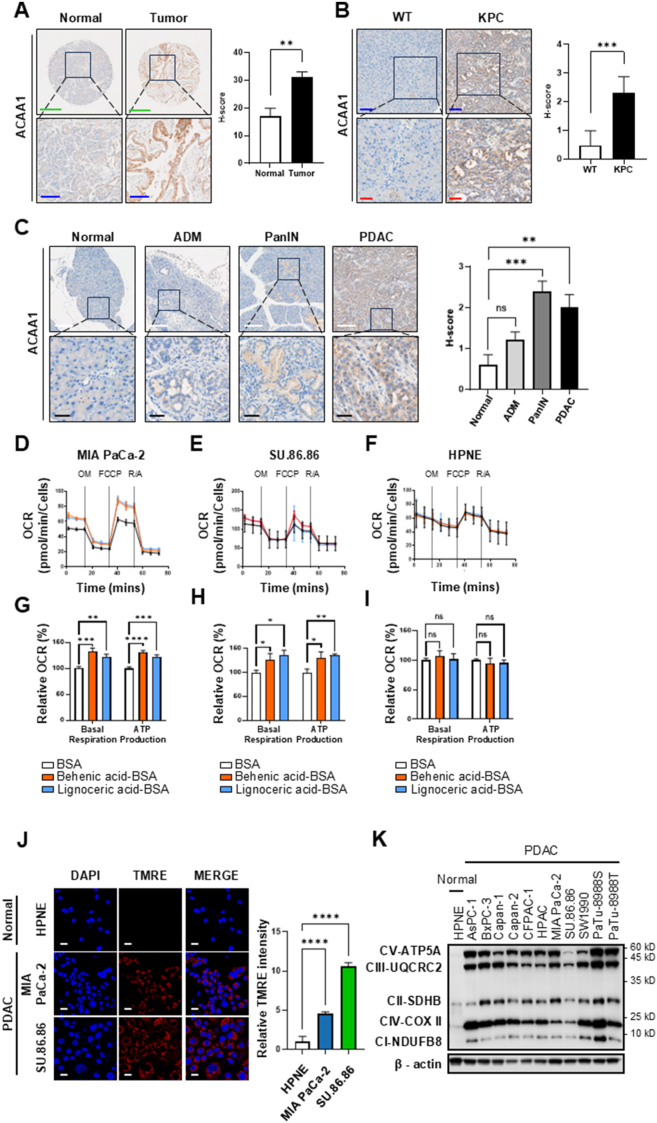
The expressions of the peroxisomal FAO key enzymes were increased in PDAC. (**A**) Immunohistochemical staining of ACAA1 was performed using a tissue microarray derived from normal tissue (n = 27) and PDAC tissue (n = 175). Scale bar (green = 400 μm, blue = 100 μm). (**B**) Representative immunohistochemical staining images of ACAA1 in wild-type (WT) and KPC mice. Scale bar (blue = 100 μm, red = 50 μm). (**C)** Representative immunohistochemical images showing ACAA1 expression at different stages of pancreatic cancer progression in KPC mice. Progressive increase in ACAA1 staining is observed in acinar-to-ductal metaplasia (ADM), pancreatic intraepithelial neoplasia (PanIN), and pancreatic ductal adenocarcinoma (PDAC). Scale bar (white = 200 μm, black = 40 μm). (**D-I**) Basal respiration and ATP production were evaluated using a Seahorse XFe analyzer in normal and cancer cells after a 3 h incubation with 20 μM BSA-conjugated VLCFAs. (Behenic acid-BSA = C22:0, Lignoceric acid-BSA = C24:0). OM, 1 μM Oligomycin; FCCP, 1 μM Carbonyl cyanide-4-(trifluoromethoxy)phenylhydrazone; and Rot/Ant, 1 μM Rotenone and 1 μM Antimycin A. Relative OCR for basal respiration and ATP production, defined as the oxygen consumption rate normalized using values derived from the SRB assay. (**J**) The mitochondrial membrane potential in HPNE, MIA PaCa-2, and SU.86.86 cells were measured by fluorescence microscopy following a 30 min TMRE staining. Scale bar (white = 20 μm). Scale bar (white = 20 μm). (**K**) The expression levels of OxPhos components in normal and PDAC cells were analyzed by western blotting. IHC image data are presented as mean ± Standard Error of the Mean. OCR (n = 3) and TMRE image analysis Data (n = 4) are presented as mean standard deviation. ∗p < 0.05, ∗∗p < 0.01, ∗∗∗p < 0.001, ∗∗∗∗p < 0.0001, ns: not significant (n = 3).

In normal cells, FAO mainly occurs in mitochondria and primarily targets long-chain fatty acids. However, when *CAC* was knocked down to inhibit mitochondrial FAO in pancreatic cancer cells, ATP synthesis dropped by 40%–50% and did not decrease further [19]. Therefore, we proposed that, in addition to mitochondrial FAO, peroxisomal FAO is activated in cancer cells. To explore the impact of FAO activity in peroxisomes on pancreatic cancer, we compared the expression of ACAA1 and peroxisomal acyl-coenzyme A oxidase (ACOX1), the key proteins involved in FAO within peroxisomes, using commercially available pancreatic cancer tissue microarrays (Supplementary Fig. 1A–C). The average H-score of ACAA1 in normal tissue was 17, whereas in pancreatic cancer tissue it was 31.2, indicating a 1.83-fold increase compared to normal tissue (Figure 1A). The average H-score of ACOX1 in normal tissue was 3.7, while in pancreatic cancer tissue it was 19.4, showing a 5.24-fold increase relative to normal tissue (Supplementary Fig. 1D). The expression of ACAA1 and ACOX1, target proteins of peroxisomal FAO, was significantly increased in pancreatic cancer tissue. In an animal model, we performed immunofluorescence staining of the pancreas in KPC mice, a genetically engineered mice model for pancreatic cancer. We compared the expression of the peroxidase ACAA1 and ACOX1 with that in wild-type mice (Supplementary Fig. 1F and G). ACAA1 expression in KPC mice was 4.26-fold higher than in wild-type mice (Figure 1B). To examine changes in ACAA1 expression during pancreatic cancer development, we analyzed its expression at key stages of tumor progression in wild-type and KPC mice (Figure 1C). Images used in Figure 1C are highlighted with red boxes in Supplementary Fig. 1H. ACAA1 levels were minimal in normal pancreatic tissue but increased to 1.2-fold, 2.4-fold, and 2-fold during acinar-ductal epithelial degeneration (ADM), pancreatic intraepithelial neoplasia (PanIN), and pancreatic ductal adenocarcinoma (PDAC) stages, respectively, showing a progressive rise during tumor development. Additionally, in KPC mice at the PDAC stage, ACOX1 expression was 5.92 times higher than in wild-type mice (Supplementary Fig. 1E). We observed increased expression of key factors involved in FAO during PDAC development in the mouse model. To test whether the functional activity of peroxisomes increased in pancreatic cancer cells, we performed OCR analysis after treating the cells with BSA-conjugated very long-chain fatty acids (VLCFAs) for 3 h, noting that VLCFA oxidation occurs solely in peroxisomes [20]. When treating the pancreatic cancer cell line MIA PaCa-2 with VLCFA (behenic acid; C22 or lignoceric acid; C24), OCR increased by 1.32-fold or 1.22-fold, respectively, and ATP production increased by 1.45-fold or 1.27-fold, respectively, compared to the control group (0 time) at 3 h (Figure 1D,G). Similarly, when the pancreatic cancer cell line SU.86.86 was treated with VLCFA (behenic acid; C22 or lignoceric acid; C24) for 3 h, OCR increased by 1.25-fold or 1.35-fold, respectively, and ATP production increased by 1.29-fold or 1.35-fold, respectively, compared to the control group (0 time) (Figure 1E,H). Acetyl-CoA produced through FAO of VLCFA contributes to OxPhos. However, normal cells (HPNE) only use long-chain fatty acids for FAO, so treating HPNE with VLCFAs such as behenic acid (C22) and lignoceric acid (C24) did not affect basal respiration or ATP production (Figure 1F,I).

To compare mitochondrial membrane potential activity between HPNE cells and cancer cells, TMRE staining was performed (Figure 1J). As a result, mitochondrial membrane potential activity was significantly higher in pancreatic cancer cell lines than in HPNE cells, with 4.51-fold higher in MIA PaCa-2 and 10.56-fold higher in SU.86.86 than in normal cells (Figure 1J). Acetyl-CoA generated from FAO produces NADH through the TCA cycle, which passes through the electron transfer complex (ETC) and generates ATP. Therefore, to assess whether increased FAO in pancreatic cancer cells leads to increased OxPhos, we examined ETC complexes I to V using western blotting (Figure 1K). In all pancreatic cancer cell lines, expression levels of OxPhos components of complexes I through V were significantly higher than those in HPNE. These results are consistent with the higher mitochondrial membrane activity observed in pancreatic cancer cells compared to normal cells, indicating increased activity due to elevated FAO.

### 3.2 *ACAA1* knockdown suppressed cell growth and decreased ATP production

**Figure 2:**
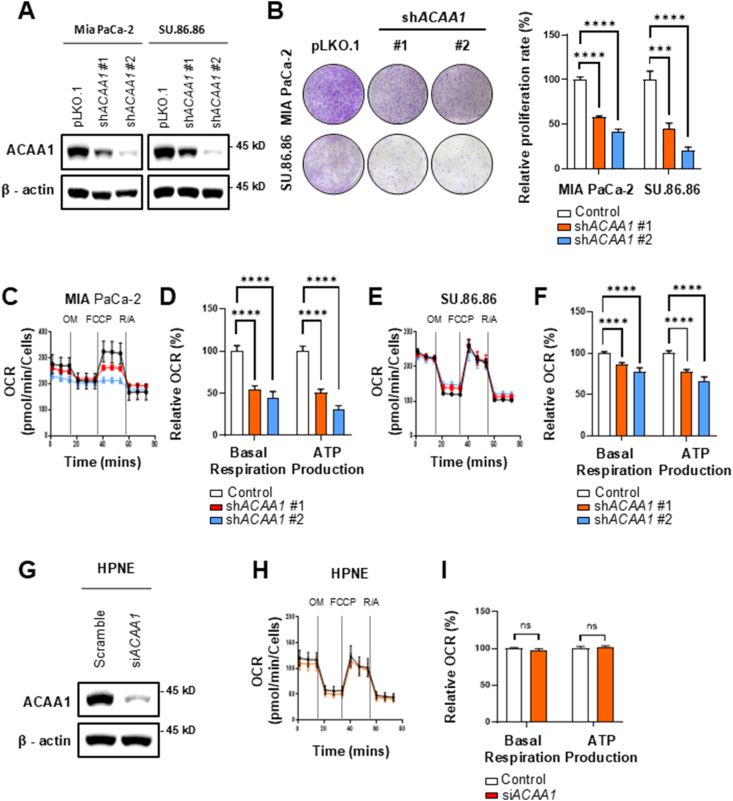
*ACAA1* knockdown suppressed cancer cell proliferation along with decreased ATP production. **(A)** The *ACAA1* knockdown using shRNA was confirmed by western blotting. **(B)** To evaluate the proliferative capacity of pancreatic cancer cells, a colony formation assay compared control (pLKO.1) cells to *ACAA1* knockdown cells over 14 days (n = 3). **(C–F)** The oxygen consumption rate (OCR) was measured using a Seahorse XFe analyzer in control and *ACAA1* knockdown PDAC cell lines (n = 6). **(G)***ACAA1* knockdown was conducted in normal cells (HPNE) using siRNA. **(H–I)** HPNE cells did not impact basal respiration and ATP production (n = 3). Data are presented as mean ± standard deviation. ∗∗∗p < 0.001, ∗∗∗∗<0.0001, ns: not significant.

To determine whether cancer cells rely on peroxisomal FAO, we performed *ACAA1* knockdown using sequences from two shRNAs in two pancreatic cancer cell lines. Knockdown efficacy was confirmed by Western blot (Figure 2A). A colony formation assay evaluated the impact of *ACAA1* knockdown on cell growth. *ACAA1* knockdown suppressed colony formation in MIA PaCa-2 by 41.5% and 58.4%, respectively, and in SU.86.86 by 56.3% and 79.7% (Figure 2B). Furthermore, ATP production decreased following *ACAA1* knockdown in MIA PaCa-2 by 49.1% and 69.4% (Figure 2C,D), and in SU.86.86 by 22.3% and 33.3% (Figure 2E,F). To assess effects on normal cells, we used siRNA to knock down *ACAA1* in HPNE cells (Figure 2G). In normal cells, *ACAA1* knockdown did not alter basal respiration or ATP production (Figure 2H,I). In various other cancer cell lines besides pancreatic cancer, including OVCAR-8 (ovarian cancer), A549 (lung cancer), SNU-449 (liver cancer), HCT-8 (colon cancer), and PC-3 (prostate cancer), *ACAA1* knockdown similarly reduced basal respiration and ATP production (Supplementary Fig. 2A and B). These findings suggest that FAO inhibition may have cancer cell-specific effects in restricting growth and energy production across different carcinomas.

### 3.3 Analysis of metabolic changes caused by *ACAA1* knockdown in cancer cells

**Figure 3:**
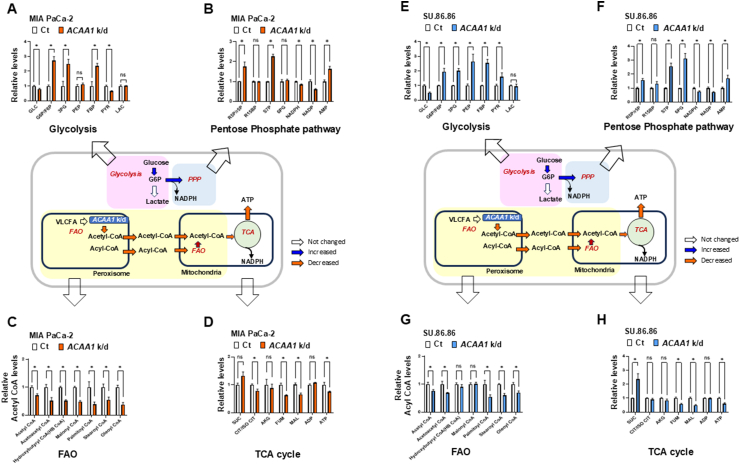
Metabolic pathway analysis of MIA PaCa-2 **(A**–**D)** and SU.86.86 cells **(E**–**H)** under *ACAA1 shRNA* knockdown using targeted LC-MS/MS. **(A and E)** Metabolites from the glycolysis pathway. **(B and F)** Metabolites from the pentose phosphate pathway. **(C and G)** Metabolites derived from acyl CoA. **(D and H)** Metabolites from the TCA cycle pathway. All metabolite levels were normalized using a BCA protein assay. (n = 3) Data are shown as mean ± standard deviation. ∗p < 0.05, ns: not significant. (white bars = Control, red bars = *ACAA1* knockdown.).

To investigate the effect of *ACAA1* knockdown on various intracellular metabolic processes, LC-MS/MS analysis was conducted. In the MIA PaCa-2 and SU.86.86 cell lines, lactate levels remained unchanged, although there was an increase in glycolytic intermediates not directly related to *ACAA1* knockdown (Figure 3A,E). In the pentose phosphate pathway (PPP), no significant changes were observed except for a notable rise in sedoheptulose-7-phosphate (S7P, a key intermediate in the non-oxidative phase of the PPP) (Figure 3B,F). Additionally, reductions were detected in acyl-CoA derivatives, including acetyl-CoA and palmitoyl-CoA (Figure 3C,G). In the TCA cycle, intermediates such as malate and fumarate also decreased (Figure 3D,H). Furthermore, *ACAA1* knockdown caused a decrease in ATP and an increase in AMP levels (Figure 3B,D, F, H). This analysis suggests that peroxisomal *ACAA1* knockdown impairs acyl-carnitine metabolism in FAO pathways, thereby disrupting energy metabolism.

### 3.4 The *ACAA1* knockdown-induced autophagy

ATP production decreased by 22.3%–69.4% following *ACAA1* knockdown (Figure 2D,F). This aligns with the 35–60% reduction in ATP production observed when we knocked down CAC in mitochondria [19]. This suggests that a significant portion of ATP production in cancer cells depends on peroxisomal FAO. We examined whether *ACAA1* knockdown decreases ATP production and leads to mTOR inactivation (Supplementary Fig. 3A and B). When *ACAA1* was knocked down for 48 h using siRNA in MIA PaCa-2 cells, mTOR activity was reduced by up to 2-fold (Supplementary Fig. 3A and B). These findings align with the observed reduction in ATP synthesis and mTOR signaling inactivation following *CAC* knockdown in mitochondria [19]. Since mTOR activation inhibits autophagy, its inactivation leads to autophagy activation. Therefore, *ACAA1* knockdown increased the LC3-II expression by up to 3.5-fold and 3.2-fold, respectively, in MIA PaCa-2 and SU.86.86 cells, whereas mTOR was inactivated by up to 2-fold (Supplementary Fig. 3A and B). This suggests that peroxisomal FAO significantly contributes to ATP synthesis in cancer cells, whereas ATP production in normal cells relies on glucose oxidation.

**Figure 4:**
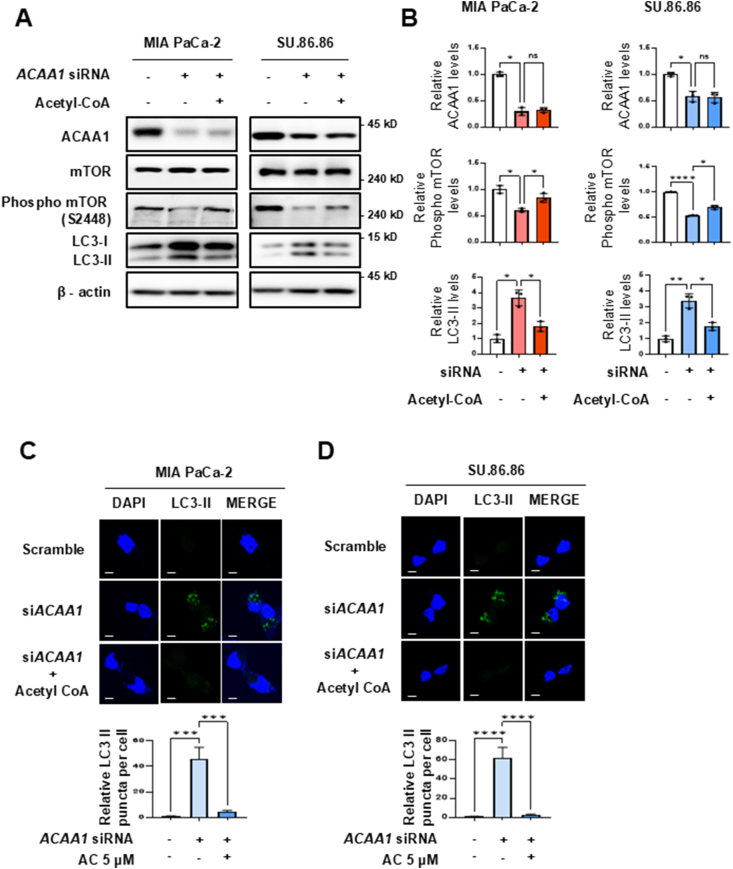
Acetyl-CoA treatment in PDAC cells with *ACAA1* knockdown rescued mTOR activation. **(A and B)** After *ACAA1* knockdown, acetyl-CoA treatment rescues mTOR phosphorylation activity and suppresses autophagy in PDAC cells. The intensity of each band was quantified using ImageJ software. **(C and D)** Representative confocal images of LC3-II immunofluorescence in control, *ACAA1* knockdown for 48 h, and *ACAA1* knockdown with 5 μM acetyl-CoA rescue for 24 h. Quantification of the number of LC3 puncta was performed using ImageJ. Scale bar (white = 20 μm). Data are presented as mean ± standard deviation. ∗p < 0.05, ∗∗p < 0.01, ∗∗∗p < 0.001, ∗∗∗∗p < 0.0001, ns: not significant.

*ACAA1* knockdown reduced acetyl-CoA production, the final product of FAO, leading to decreased ATP levels. Therefore, we investigated whether supplementing acetyl-CoA after *ACAA1* knockdown could restore mTOR activation, as shown by western blotting (Figure 4A,B). In MIA PaCa-2 cells, adding acetyl-CoA following *ACAA1* knockdown resulted in a 1.4-fold increase in mTOR phosphorylation. Additionally, the LC3-II decreased by 2-fold (Figure 4A,B). In SU.86.86 cells, after ACAA1 knockdown, acetyl-CoA supplementation caused a 1.3-fold increase in mTOR phosphorylation. The LC3-II decreased by 1.9-fold (Figure 4A,B). These findings indicate that ATP production declined due to FAO defects caused by *ACAA1* knockdown, which consequently led to inactivation of p-mTOR and activation of autophagy. To assess ACAA1's role in autophagy regulation, LC3-II immunofluorescence was performed in MIA PaCa-2 and SU.86.86 cells using siRNA-mediated *ACAA1* knockdown. Confocal microscopy revealed that in MIA PaCa-2 cells, LC3-II puncta increased by 45.2-fold in *ACAA1* knockdown cells compared to controls (Figure 4C), and by 61.5-fold in SU.86.86 cells (Figure 4D). After treating the cells with *ACAA1* siRNA for 48 h and supplementing with acetyl-CoA for 24 h, the levels returned to normal (Figure 4D). These results suggest that inhibiting FAO through *ACAA1* knockdown induced autophagy activation, which was reversed by supplementing acetyl-CoA, the final product of FAO.

### 3.5 ACAA1 inhibition, either through *ACAA1* knockdown or with an ACAA1 inhibitor, decreased tumor development in an animal model

**Figure 5:**
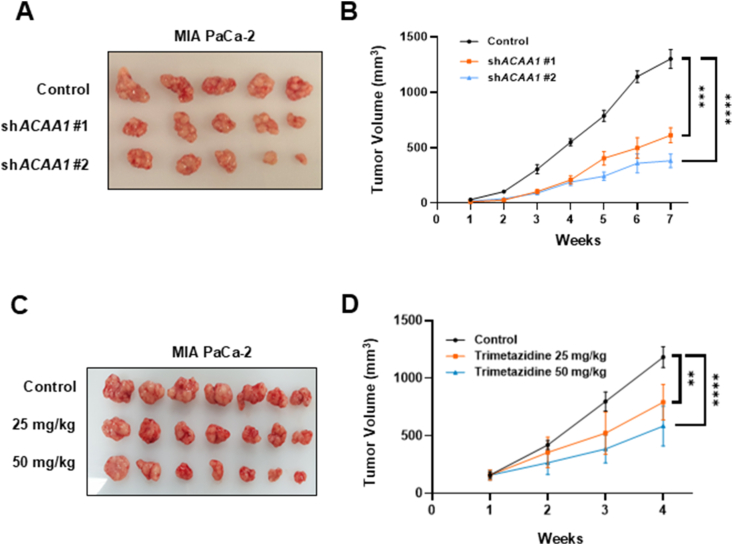
Blocking ACAA1 activity by *ACAA1* knockdown or ACAA1 inhibitor significantly reduced pancreatic cancer development in animal models. (**A**) A representative image of tumors derived from control and *ACAA1* knockdown MIA PaCa-2 cells. 1 × 10^7^ Control and *ACAA1* knockdown cells were subcutaneously injected into BALB/c-nude mice (n = 5, 8 weeks old). (**B**) Tumor size was measured weekly using calipers since the tumor was seeded (0–30 mm^3^, with the first day of measurement designated as 1 w). Tumor volume was calculated using the following formula: V = (A × B^2^)/2, where V is the volume (mm^3^), A is the long diameter, and B is the short diameter. (**C**) Representative image of tumors derived from control and trimetazidine-treated (an ACAA1 inhibitor [21]) groups in MIA PaCa-2 xenograft models. (**D**) Once tumor volume reached approximately 100 mm^3^ (at 2.5 w since tumor was seeded), mice were randomly assigned to groups designated as a 1 w. Trimetazidine was administered orally at doses of 25 or 50 mg/kg (5 days a week). Tumor volume was calculated using the following formula: V = (A × B^2^)/2, V is the volume (mm^3^), A is the long diameter, and B is the short diameter. Data are presented as mean ± standard deviation. ∗∗p < 0.01, ∗∗∗p < 0.001, ∗∗∗∗p < 0.0001, and ns: not significant.

To assess the impact of ACAA1 on tumor growth in xenograft models, *ACAA1* shRNA was transfected into the MIA PaCa-2 cell line to suppress ACAA1 expression. The cells were then transplanted into 8-week-old nude mice. After transplantation, the average tumor volume in the *ACAA1* knockdown group decreased by 53%, 70%, and 7%, respectively, compared to the control group (Figure 5A,B). Additionally, MIA PaCa-2 xenograft mice were treated with different concentrations of the ACAA1 inhibitor trimetazidine for four weeks to evaluate whether it produced a similar effect. Trimetazidine has been reported to competitively inhibit ACAA1 with various substrates, including short-chain to long-chain fatty acids [21]. By week 4, tumor formation was reduced by 33% in the 25 mg/kg group and by 50.6% in the 50 mg/kg group compared to the control group (Figure 5C,D).

**Figure 6:**
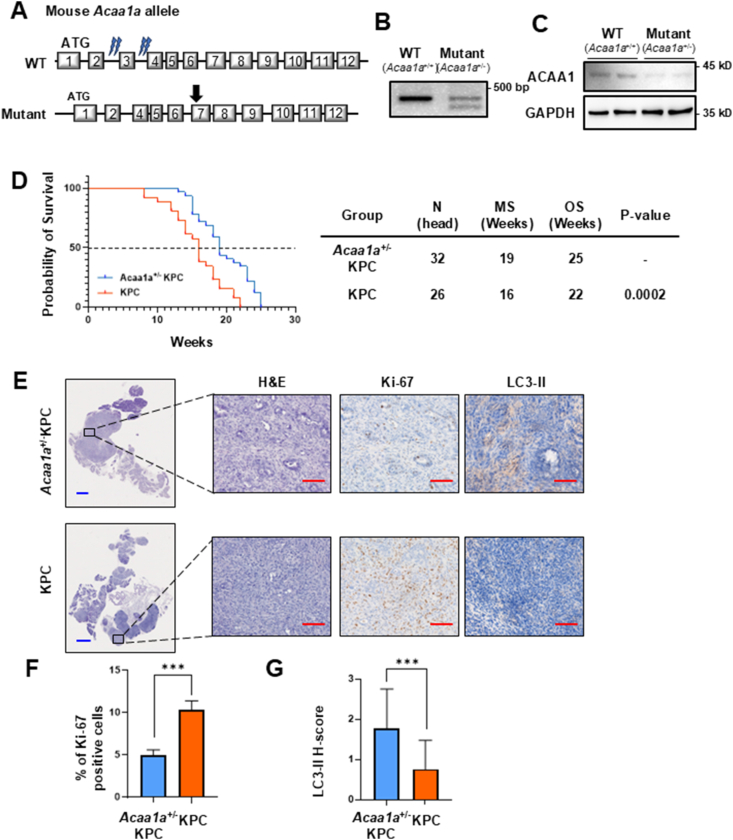
The *Acaa1a* knockout significantly reduced pancreatic cancer development in the KPC model. (**A**) Generation of *Acaa1a*-null mouse (Exon 3 deletion). Schematic representation of *Acaa1a* knockout mouse generation. The *Acaa1a* gene was targeted using the CRISPR/Cas9 system, resulting in the production of a truncated mutant protein. ATG, translational start site; black arrow, translational stop site. (**B**) The deletion of *Acaa1a* was confirmed using PCR. (**C**) The protein level was confirmed by western blotting. (**D**) Kaplan–Meier survival curves for KPC (n = 26) and *Acaa1a*^+/−^;KPC mice (n = 32). The median survival of *Acaa1a*^+/−^;KPC mice was 3 weeks longer than that of KPC mice. (**E**) Immunohistochemical staining of H&E, Ki-67, and LC3-II in *Acaa1a*^+/−^;KPC and KPC mice pancreas. (**F**) The quantification percentage of Ki-67 positive cells and (**G**) the average H-score for LC3-II were performed using the Form Image Analysis Software (Akoya Biosciences, Marlborough, MA, USA). Scale bar (blue = 2 mm, red = 100 μm). Data are presented as mean ± standard deviation. ∗∗∗p < 0.001.

*Acaa1a*^*+/−*^ mice were created using the CRISPR/Cas9 system. The Methods section provides detailed information on how the knockout mice were generated and their genotype (Figure 6A). Deletion of the *Acaa1a* gene was confirmed by PCR (Figure 6B), and reduced protein levels were validated through Western blotting (Figure 6C). To investigate whether the *Acaa1a* gene influences the developmental stages of pancreatic cancer, we crossed *Acaa1a*^*+/−*^ mice with KPC mice (*Kras*^LSL−G12D/+^, *Trp53*^LSL−R172H/+^, *Pdx1*-Cre) to produce *Acaa1a*^*+/−*^/KPC mice, a model for pancreatic cancer. The median survival (MS) of KPC mice and *Acaa1a*^*+/−*^/KPC mice was 16 and 19 weeks, respectively (Figure 6D). At 22 weeks, the survival rates of KPC and *Acaa1a*^*+/−*^/KPC mice were 0% and 34%, respectively. Immunohistochemical staining was performed on mouse tissue using antibodies to Ki-67 and LC3-II (Figure 6E and Supplementary Fig. 4). The Ki-67 positive area in *Acaa1a*^*+/−*^/KPC mice decreased by 96% compared to KPC mice (Figure 6F). The average H-Score for LC3-II expression was 2.3 times higher in *Acaa1a*^*+/−*^/KPC mice than in KPC mice (Figure 6G). These results suggest that *Acaa1a* deficiency reduces ATP production by inhibiting FAO, which promotes autophagy and ultimately suppresses tumor growth, resulting in improved survival in an animal model.

## 4 Discussion

The roles of autophagy as a tumor suppressor are proposed as some of the indirect anticancer effects, which involve mechanisms such as removing damaged organelles and proteins, and limiting cell growth and genomic instability [22,23], by restricting genome mutations and ensuring protein and organelle quality control [24,25], by protecting healthy cells and reducing inflammation [23], and by scavenging damaged oxidative organelles to prevent the accumulation of toxic oxygen radicals [26]. However, autophagy alone has limitations in explaining tumor growth inhibition. In previous studies, we observed that when autophagy was induced in cancer cells responding to anticancer drugs, mTOR was activated in those cells. This activation allowed the cells to shift from the death pathway to a survival pathway, along with autophagy [3]. This indicates that ATP production increases during the induction of FAO by autophagy in cancer cells, which then activates mTOR. Recently, we showed that inhibiting FAO via *CAC* knockdown triggered autophagy by decreasing ATP production, leading to mTOR inactivation [19].

Cancer cells can activate mTOR due to increased ATP levels, which they use to produce new biomolecules that support cell regrowth. Previous studies have shown that cancer cells largely depend on FAO for ATP production [6,7]. In this study, we found that FAO is active in mitochondria and peroxisomes, contributing to nearly 50% of ATP production in cancer cells. Knockdown of *ACAA1* in peroxisomes showed that mTOR was inactivated. Peroxisome FAO is likely essential due to the variety of substrates in cancer. Mitochondria mainly handle the oxidation of long-chain fatty acids, while peroxisomes facilitate β-oxidation of various fatty acids, including not only long- and medium-chain fatty acids released from lipids but also very long-chain fatty acids, free fatty acids, dihydroxycholesterol acid, and trihydroxycholesterol acid, which are intermediates of bile acids [27]. Therefore, inhibiting peroxisomal FAO may enhance the anti-cancer effect when combined with mitochondrial FAO inhibition by reducing ATP production. Metabolites from peroxisomal FAO are transported to mitochondria for further processing in both normal and cancer cells [28]. Reports showed that peroxisomal FAO is increased in various cancer types, including liver, breast, melanoma, and colon cancers [[29], [30], [31]], as well as PDAC in this study.

**Figure 7:**
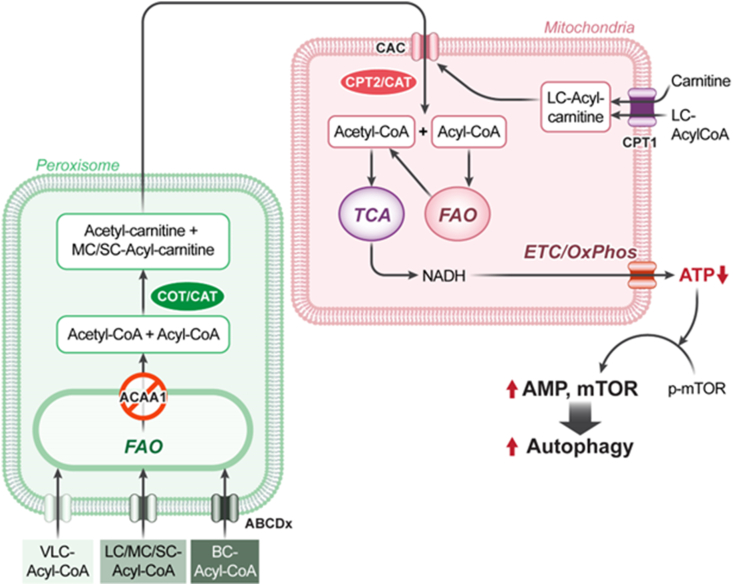
The *ACAA1* knockdown induced autophagy through the decreased ATP production. The interaction between peroxisomes and mitochondria has been well-reviewed in FAO [9]. In normal mitochondria, LC-acyl-CoA is mainly transferred by CPT1 and used as the primary energy source through FAO. In cancer mitochondria, however, MC-/SC-acyl-CoA is used as an energy source in addition to LC-acyl-CoA (Figure 3) [9,48]. The MC-/SC-acyl-CoA binds to carnitine by CAT and COT [36] and is transported to the mitochondria via CAC. The transferred acyl-carnitines are metabolized to acyl-CoAs by CPT2 (carnitine palmitoyltransferase II) [9] or CAT. Long chain-(LC-), mid-chain-(MC-), and short chain–(SC–)acyl-CoA; CAC, carnitine acyl-carnitine carrier; CAT, carnitine acetyltransferase; COT, carnitine octanoyl transferase; ETC-OxPhos; electron transfer complex-oxidative phosphorylation.

Therefore, the interaction between peroxisomes and mitochondria in FAO is a key energy production process because ATP is generated solely in the inner mitochondrial membrane using carbon sources from fatty acids delivered by the peroxisome or plasma [32]. In the functional aspects of normal cells, mitochondria efficiently metabolize long-chain fatty acids (C13∼C21) [33], while peroxisomes metabolize very long-chain fatty acids (>C21) [34] and long-chain fatty acids [35]. In cases of hereditary peroxisomal disorder, the accumulation of very long-chain and branched fatty acids indicates that peroxisomes may primarily metabolize them [9]. However, peroxisomes do not fully break down fatty acids into acetyl-CoA because of the chain-length specificities of the enzymes involved, which mainly stop at mid-chain fatty acids (C6–C12) and short-chain fatty acids (C < 6) [9]. These mid-chain fatty acids are transported to mitochondria for further breakdown. The mechanism of moving mid-chain acyl-carnitine to the cytosol is not fully understood. However, it may diffuse into the cytosol, from where it can then be transferred to the mitochondria through CAC. In the peroxisome, mid-chain acyl-CoA and short-chain acyl-CoA can be further processed into mid-chain acyl-carnitine and short-chain acyl-carnitine by carnitine O-octanoyl transferase (COT) [36] and carnitine acetyl-transferase (CAT) [37], which are actively transferred to mitochondria through CAC. Peroxisomal CAT and COT catalyze the transesterification of acyl-CoA to acylcarnitine, facilitating the metabolic exchange of acyl groups between peroxisomes and mitochondria. *CAC* knockdown caused a buildup of mid-chain acyl-carnitine and decreased ATP production in PDAC cells, indicating that abundant mid-chain acyl-carnitine is used to be processed into acetyl-CoA for ATP generation [19]. The short-chain and mid-chain acyl-carnitines are transported into mitochondria via CAC because CAC-deficient human fibroblasts showed that the peroxisome accumulates mid-chain lauric acid (C12) [38]. The long-chain acyl-carnitines are processed by carnitine palmitoyltransferase 1 (CPT1) and transferred to the CAC [39]. Therefore, the interaction between peroxisome and mitochondria in FAO plays a crucial role in ATP production in PDAC cells (Figure 7). This can be seen as a new signaling pathway that activates autophagy specifically in cancer cells. In normal cells, the primary signal for autophagy is AMPK activation caused by a lack of glucose or amino acids [40]. However, for unknown reasons, cancer cells do not use glucose as an energy source but convert it entirely into lactate, making them dependent on FAO for ATP production. In normal cells, knocking down *CAC* or *ACAA1* to inhibit FAO does not activate AMPK because glucose oxidation is essential for ATP generation. In cancer cells, however, knocking down *CAC* or *ACAA1* decreases ATP production, which then activates AMPK. Therefore, the discovery that inhibiting FAO activates autophagy in cancer cells reveals a completely new signaling pathway that was previously unknown. Additionally, the increase in autophagy caused by anticancer agents is associated with activation of mTOR [3]. Therefore, mTOR activation happens in cancer cells that become resistant to anticancer drugs after long-term treatment [[41], [42], [43], [44]]. However, in cancer cells, the reduction in ATP caused by FAO inhibition does not activate mTOR but instead induces autophagy, leading to anticancer effects. Therefore, it is expected that combining traditional cytotoxic anticancer drugs with FAO inhibitors will result in highly effective anticancer activity.

We found that increased peroxisomal FAO in PDAC is inversely related to overall survival in PDAC patients. Inhibiting FAO through *ACAA1* knockdown in a xenograft model with human PDAC cells significantly induced autophagy due to decreased ATP production. A PDAC mice KPC model with an *Acaa1a*^*+/−*^ also activated autophagy. Additionally, these two models consistently showed reduced tumor growth and improved survival rates. These findings suggest that targeting peroxisomal FAO to inhibit FAO could be a promising new therapeutic strategy for treating tumors. Although FAO inhibition with mitochondrial CPT1 inhibitors has demonstrated promising anti-cancer effects in non-clinical animal studies [45], they were discontinued because of hepatotoxicity observed in animal models and clinical trials [46,47]. Therefore, we must carefully monitor whether ACAA1 inhibition also leads to other toxicities. However, the main benefit of targeting ACAA1 is suppressing tumor growth by activating autophagy specifically in cancer cells, without harming normal cells.

## CRediT authorship contribution statement

**Ho Lee:** Writing – review & editing, Validation, Methodology, Investigation, Data curation. **Mingyu Kang:** Visualization, Validation, Methodology, Formal analysis, Data curation. **Sung Hoon Sim:** Writing – review & editing, Supervision, Methodology, Investigation, Funding acquisition. **Joon Hee Kang:** Visualization, Validation, Methodology, Formal analysis, Data curation. **Wonyoung Choi:** Writing – review & editing, Supervision, Methodology, Investigation. **Jung Won Chun:** Writing – review & editing, Supervision, Methodology, Investigation. **Woosol Hong:** Visualization, Validation, Methodology, Investigation, Data curation. **Chaeyoung Kim:** Visualization, Validation, Investigation, Formal analysis, Data curation. **Woojin Ham:** Validation, Investigation, Formal analysis. **Jeong Hwan Park:** Visualization, Validation, Investigation, Formal analysis. **Eun-Byeol Koh:** Visualization, Validation, Data curation. **Yoon Jeon:** Validation. **Sang Myung Woo:** Writing – review & editing, Methodology, Investigation. **Soo-Youl Kim:** Writing – review & editing, Writing – original draft, Project administration, Methodology, Investigation, Funding acquisition, Formal analysis, Data curation.

## Declaration of competing interest

The authors declare the following financial interests/personal relationships which may be considered as potential competing interests:Soo-Youl Kim reports administrative support was provided by NCC-Bio Co. Soo-Youl Kim reports a relationship with NCC-Bio Co. that includes: board membership. If there are other authors, they declare that they have no known competing financial interests or personal relationships that could have appeared to influence the work reported in this paper.

## Data Availability

Data will be made available on request.
