# Supplementary material for: ACAA1 knockout increases the survival rate of KPC mice by activating autophagy
Source: Mol Metab. 2025 Aug 21;100:102237. doi: 10.1016/j.molmet.2025.102237 (PMC12419109; doi:10.1016/j.molmet.2025.102237)
Supplement: Multimedia component 1 [file mmc1.pdf]

ACAA1 knockout expanded the survival rate of KPC mice through autophagy activation

Ho Lee[1,2], Mingyu Kang[1,3], Sung Hoon Sim[4], Joon Hee Kang[2,3], Wonyoung Choi[1,2,4], Jung Won Chun[2,5,7], Woosol Hong[1,3], Chaeyoung Kim[1,3], Woojin Ham[1,3], Jeong Hwan Park[1,3], Eun-Byeol Koh[1], Yoon Jeon[6], Sang Myung Woo[2,4,7] and Soo-Youl Kim[1,3]*

This file includes Supplementary Figures 1 to 4 and legends.

Supplementary Figure 1

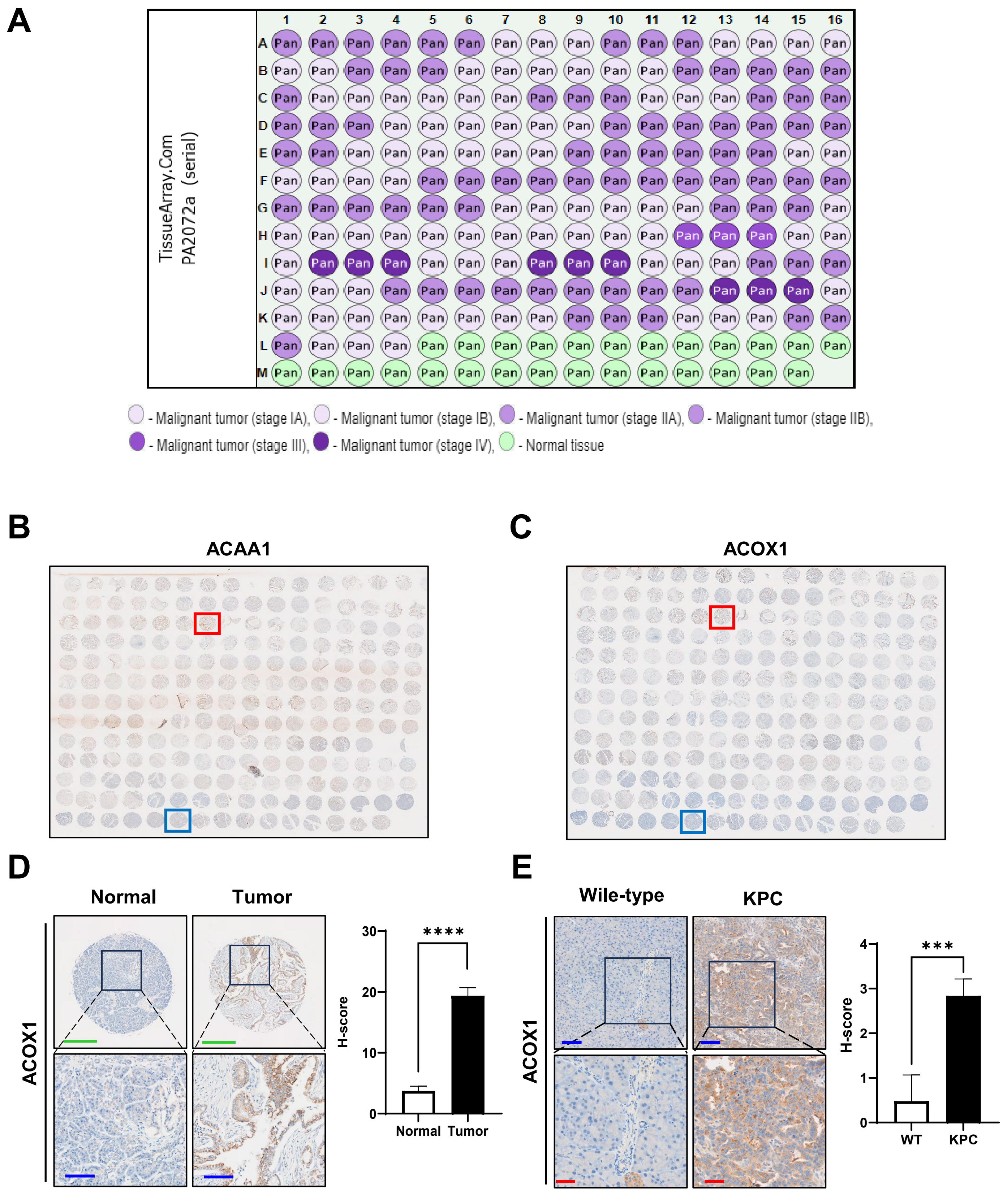

Supplementary Figure 1

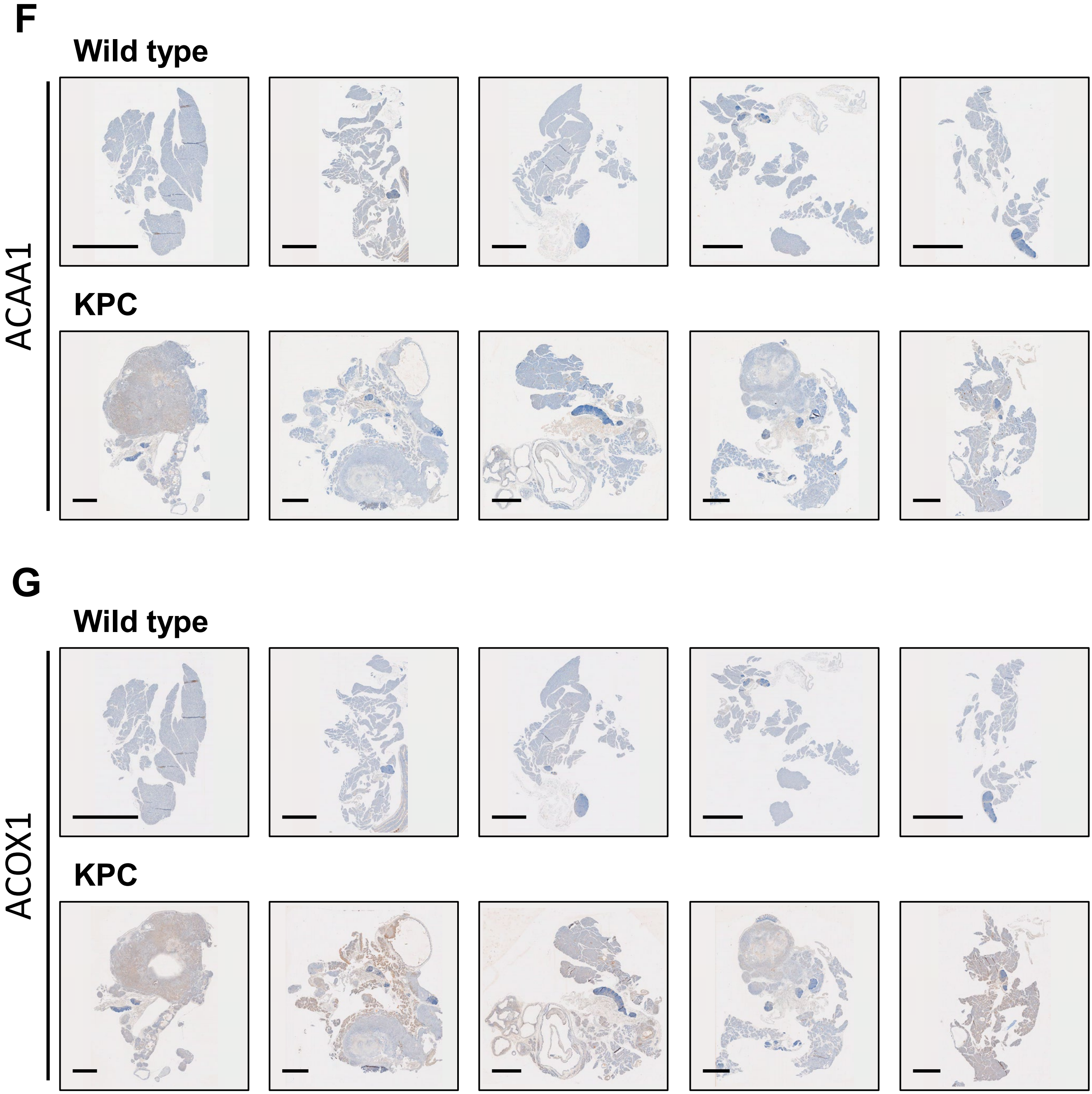

Supplementary Figure 1

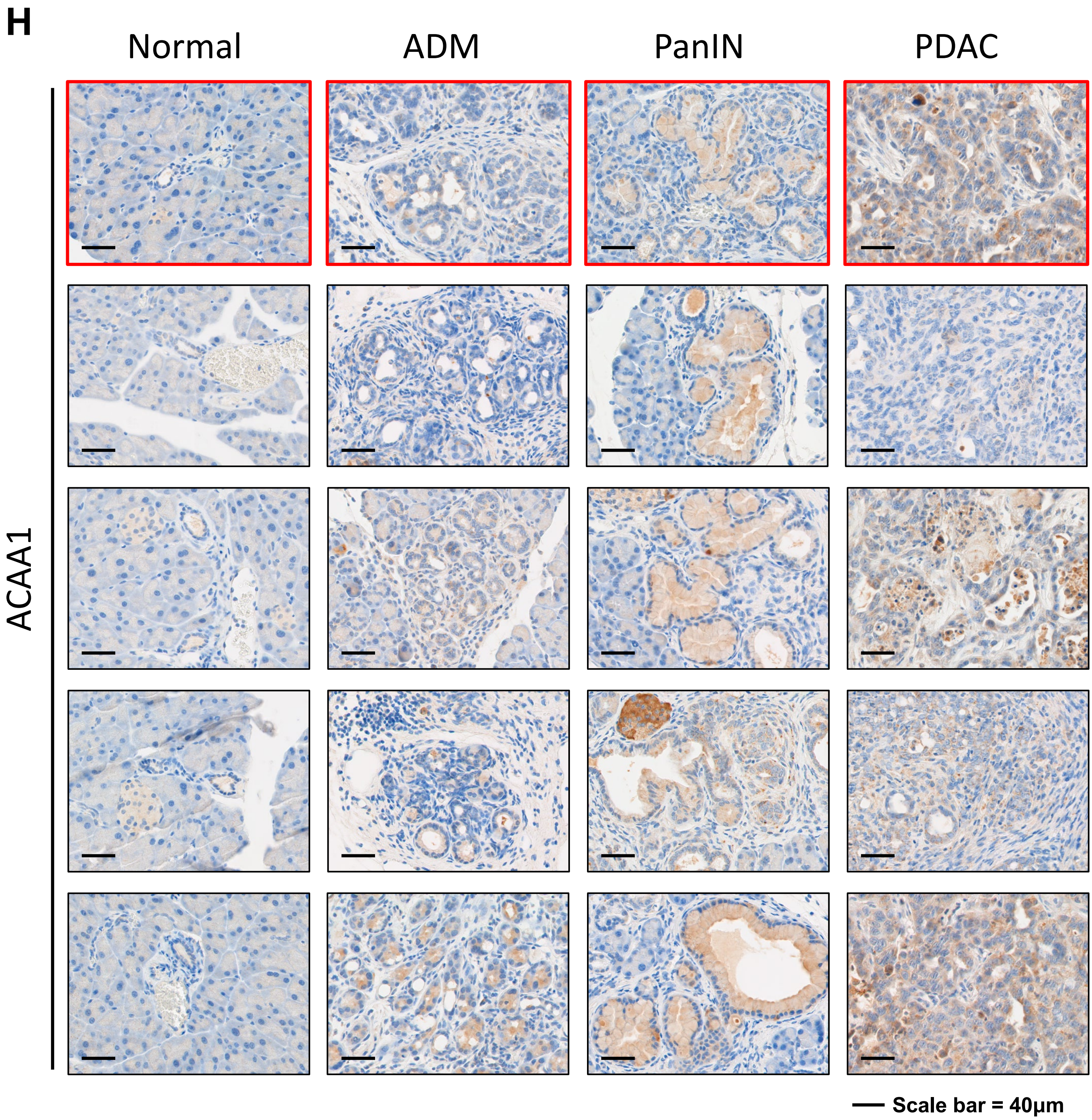

Supplementary Figure 1

Peroxisomal FAO enzymes expression in PDAC TMA. (**A**) Pancreatic carcinoma tissue microarray (TMA) tissue samples derived from normal tissue (n = 27) and PDAC tissue (n = 175) were purchased from Tissue Array (PA2072a, Derwood, MD, US). (**B-C**) Immunohistochemical staining images of above TMA (PA2072a) stained with anti-ACAA1 and anti-ACOX1 antibodies. (**D** Representative images of ACOX1 immunohistochemical staining from the TMA dataset are shown in Supplementary Fig. 1C. (Scale bar: green = 400 μm, blue = 100 μm). (**E**) Representative immunohistochemical staining images of ACOX1 in wild-type and KPC mice. Consistent with Supplementary Fig. 1D, ACOX1 expression is increased in pancreatic cancer tissues compared to normal tissues. (Scale bar: blue = 100 μm, red = 50 μm) Images correspond to Supplementary Fig. 1G. (**F-G**) Whole immunohistochemical staining images of ACAA1 and ACOX1 in pancreatic tissues from wild-type and KPC mice. Images correspond to Fig. 1C-D. ACAA1 and ACOX1 staining were used to assess peroxisomal fatty acid oxidation. (n = 5, scale bar = 3 mm). (**H**) Immunohistochemical staining images of ACAA1 in pancreatic tissues from wild-type B6 and KPC mice. Normal, ADM (acinar-to-ductal metaplasia), PanIN (pancreatic intraepithelial neoplasia), and PDAC (pancreatic ductal adenocarcinoma) lesions are each shown with five representative images in Supplementary Fig. 1F. Images used in Fig. 1C are highlighted with red boxes. ACAA1 expression is increased in pathological lesions compared to normal pancreatic tissue. (scale bar = 40 μm)

Supplementary Figure 2

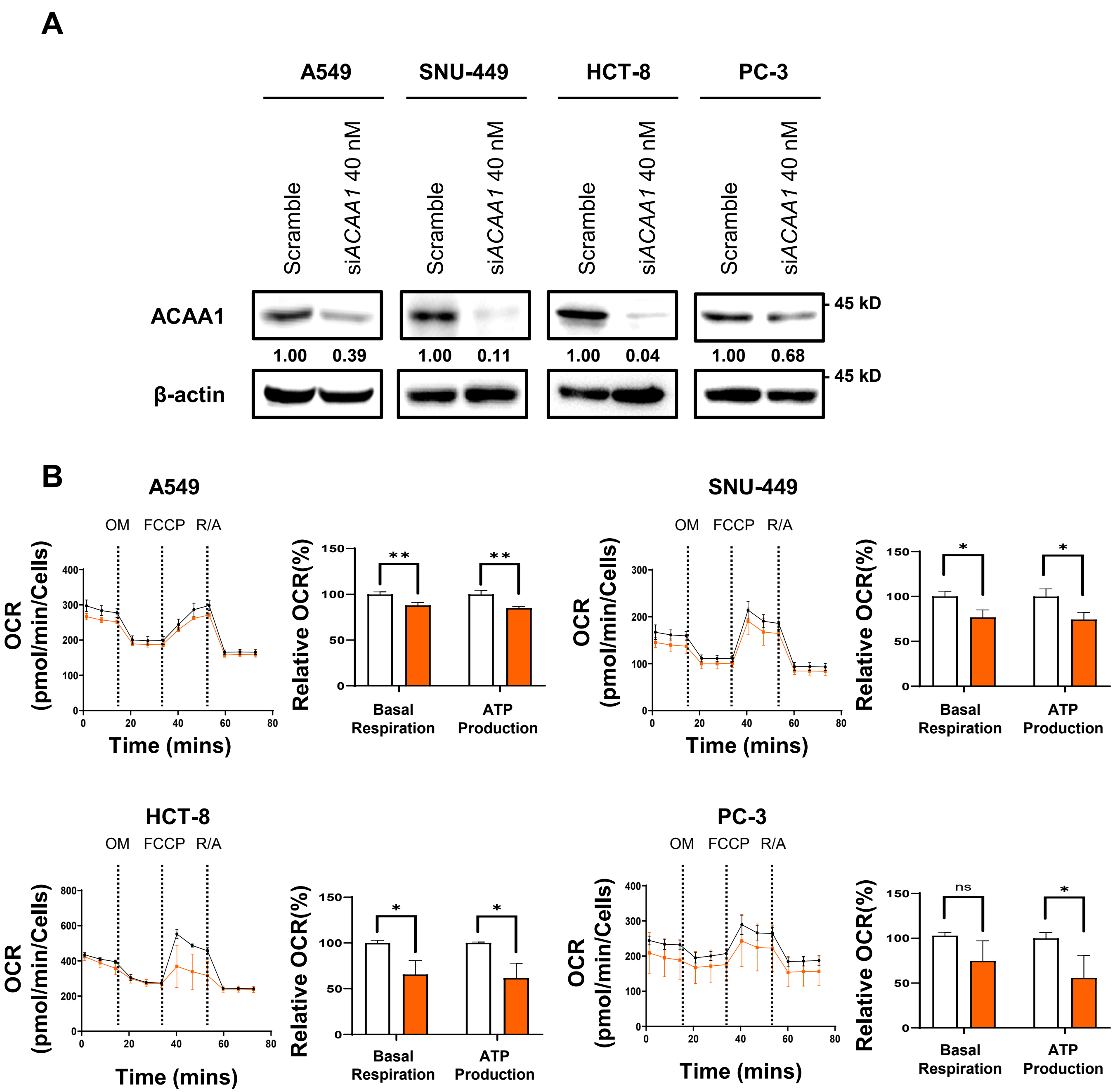

Supplementary Figure 2

Effect of ACAA1 knockdown on basal respiration and ATP production in various cancer cells. (**A**) The siRNA (40 nM for 48 h) induced knockdown of ACAA1 was confirmed by western blotting. (A549: lung cancer, SNU-449: liver cancer, HCT-8: colon cancer, PC-3: prostate cancer). (**B**) The oxygen consumption rate (OCR) was measured using a Seahorse XFe analyzer in control and ACAA1 knockdown in various cancer cell lines ($n = 3$). OM, 1 μM Oligomycin; FCCP, 1 μM Carbonyl cyanide-4-(trifluoromethoxy)phenylhydrazone; and Rot/Ant, 1 μM Rotenone and 1 μM Antimycin A. Data are presented as mean ± standard deviation. *$p < 0.05$, ** $p < 0.01$, ***$p < 0.001$, ns: not significant.

Supplementary Figure 3

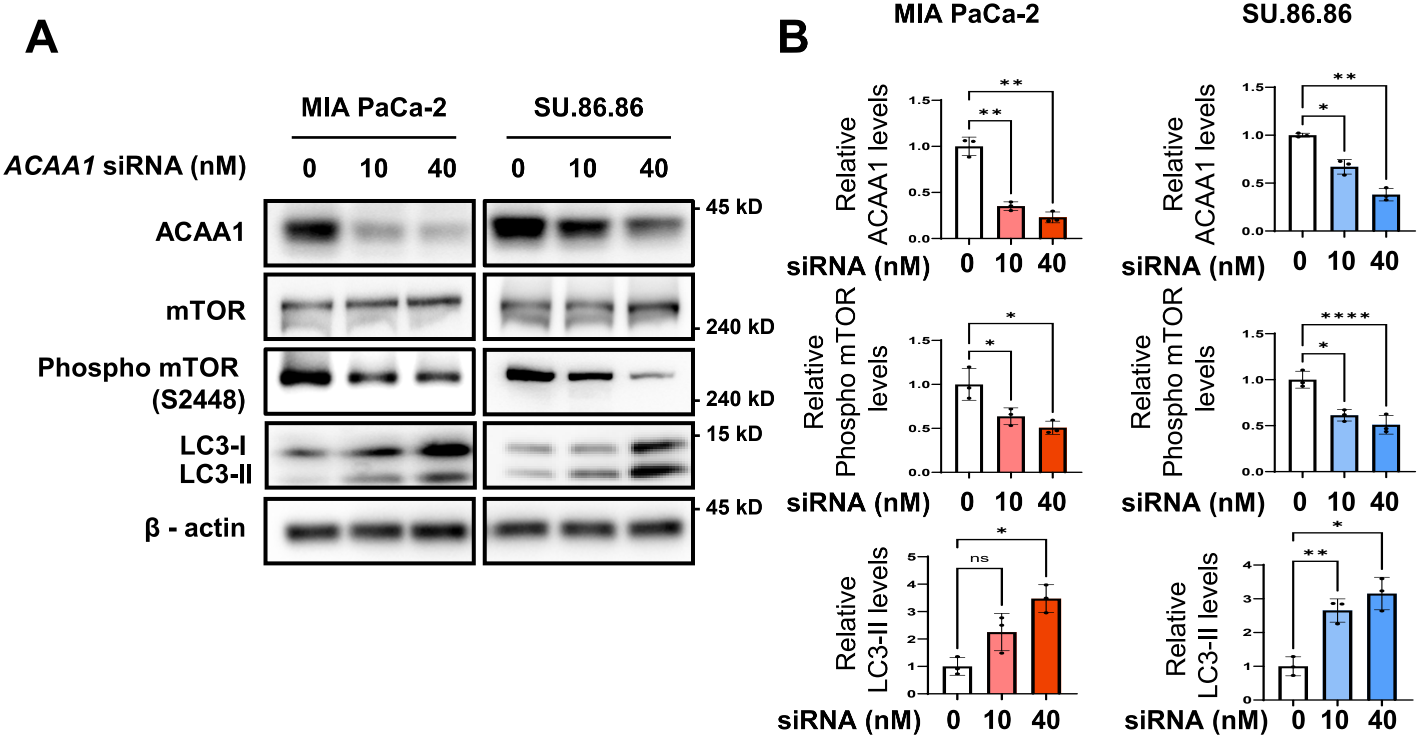

Supplementary Figure 3

The knockdown of *ACAA1* induces autophagy by suppressing mTOR phosphorylation. (**A, B**) *ACAA1* knockdown modulates mTOR phosphorylation, contributing to the induction of autophagy in PDAC cells. Data are presented as mean ± standard deviation. *p < 0.05, ** p < 0.01, ***p < 0.001, ****p<0.0001, ns: not significant.

Supplementary Figure S4

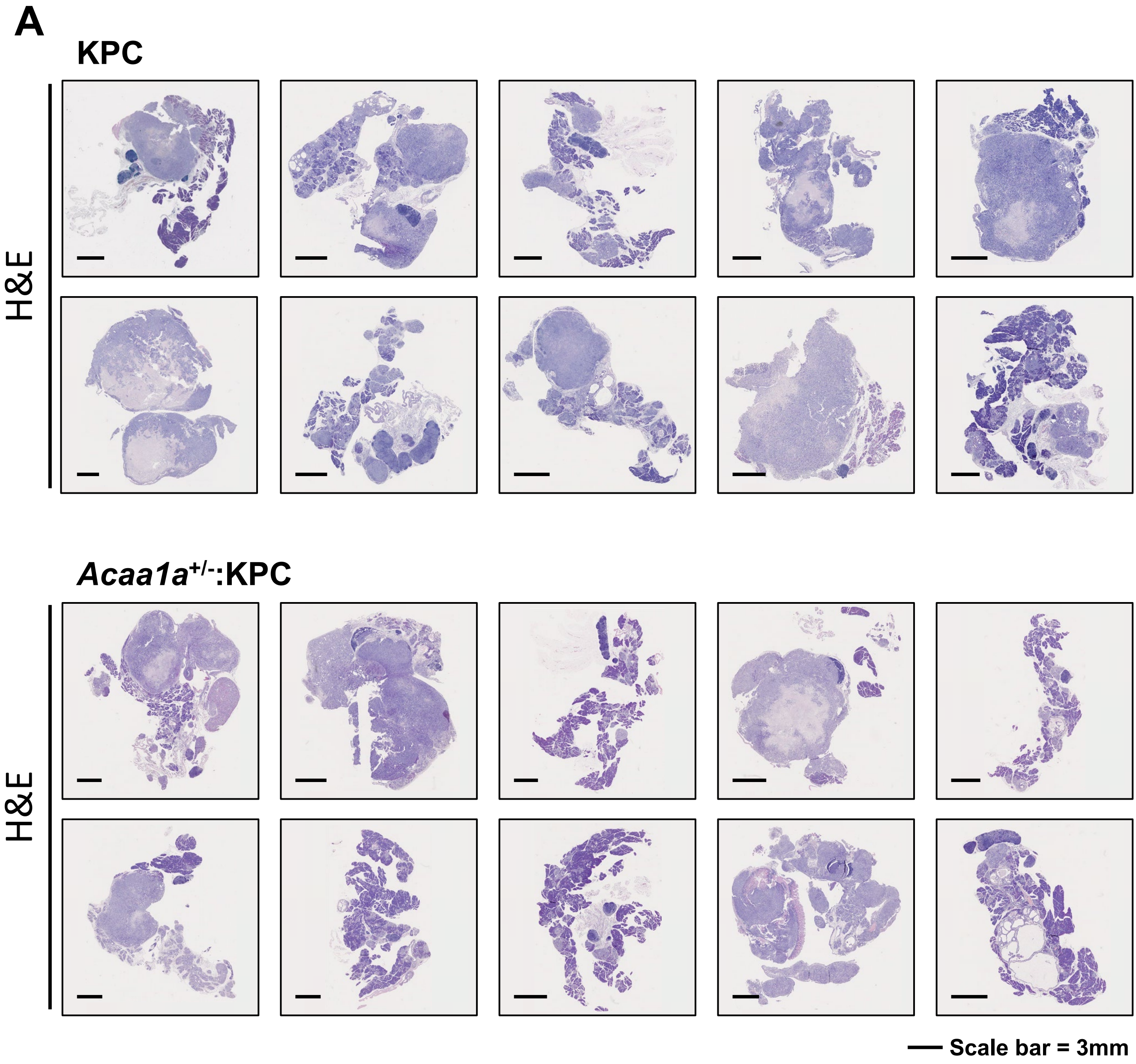

Supplementary Figure S4

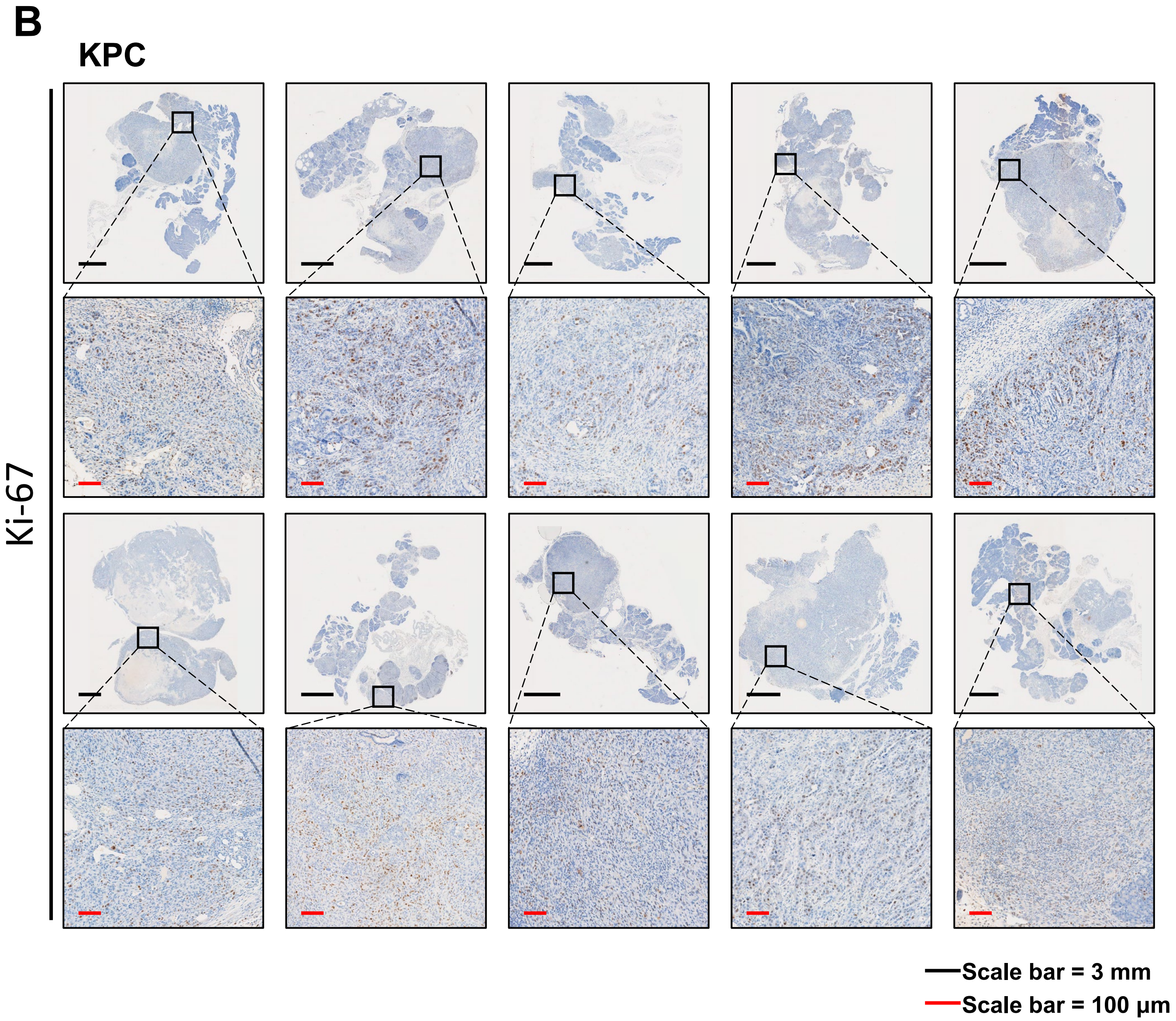

Supplementary Figure S4

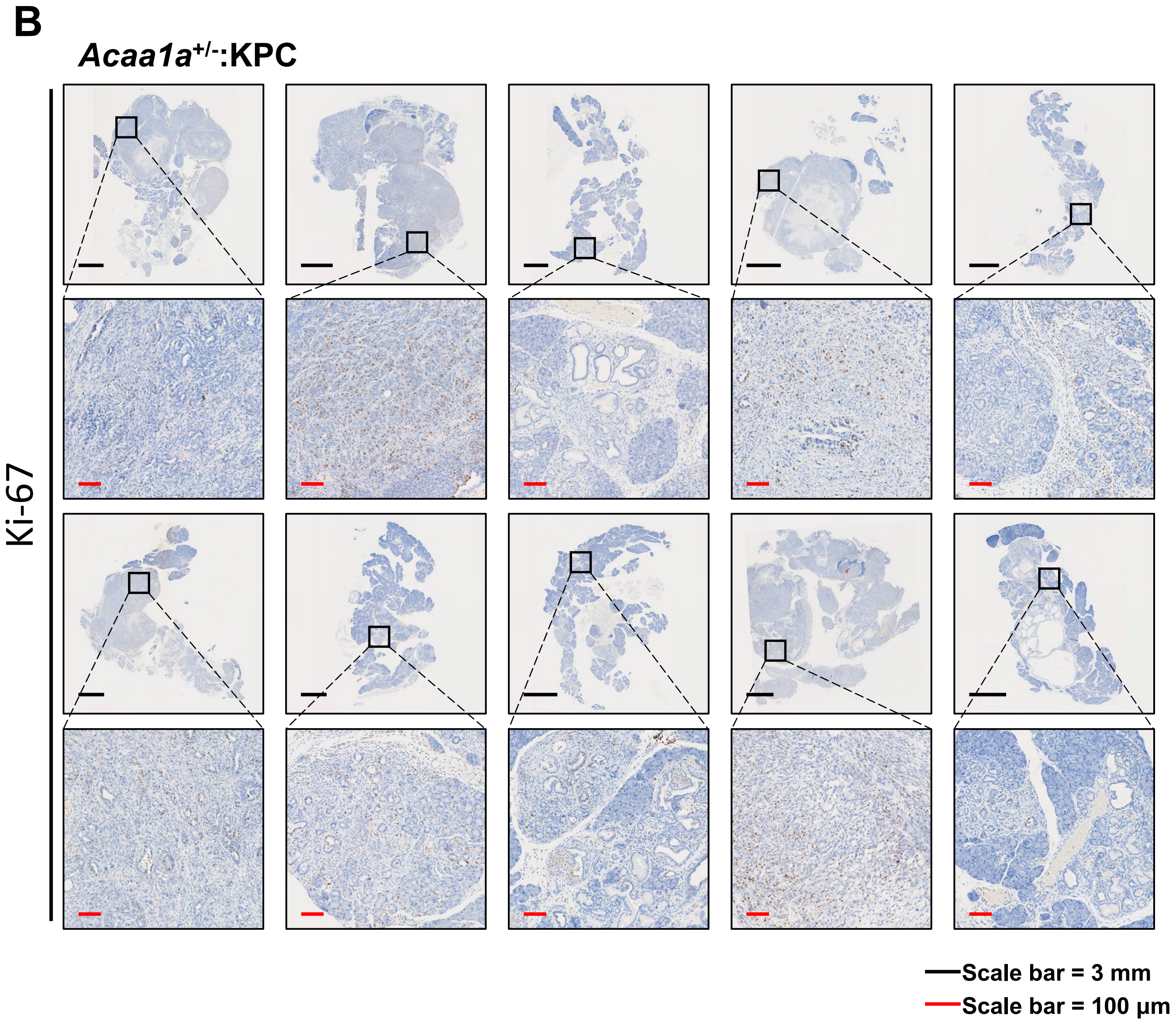

Supplementary Figure S4

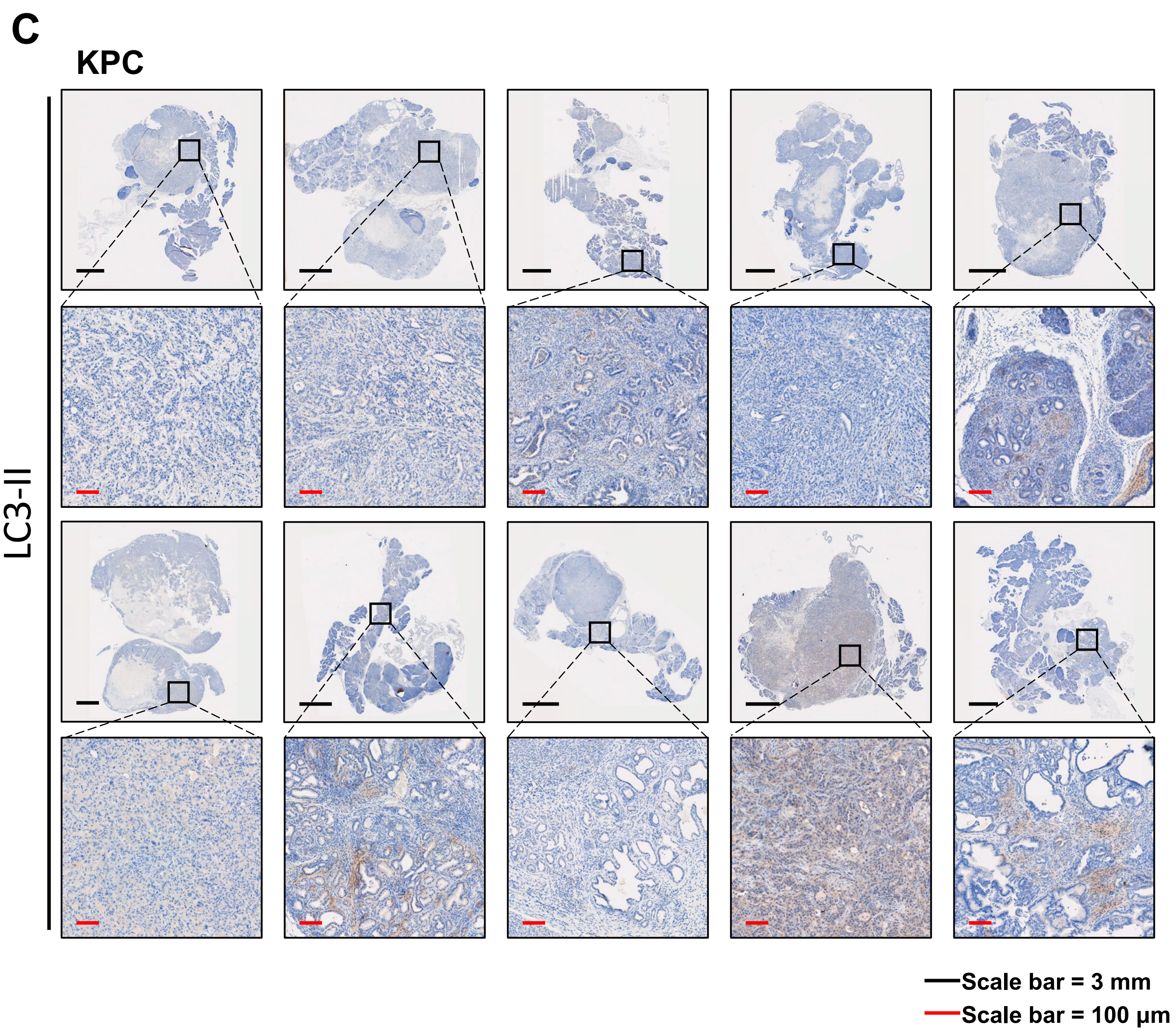

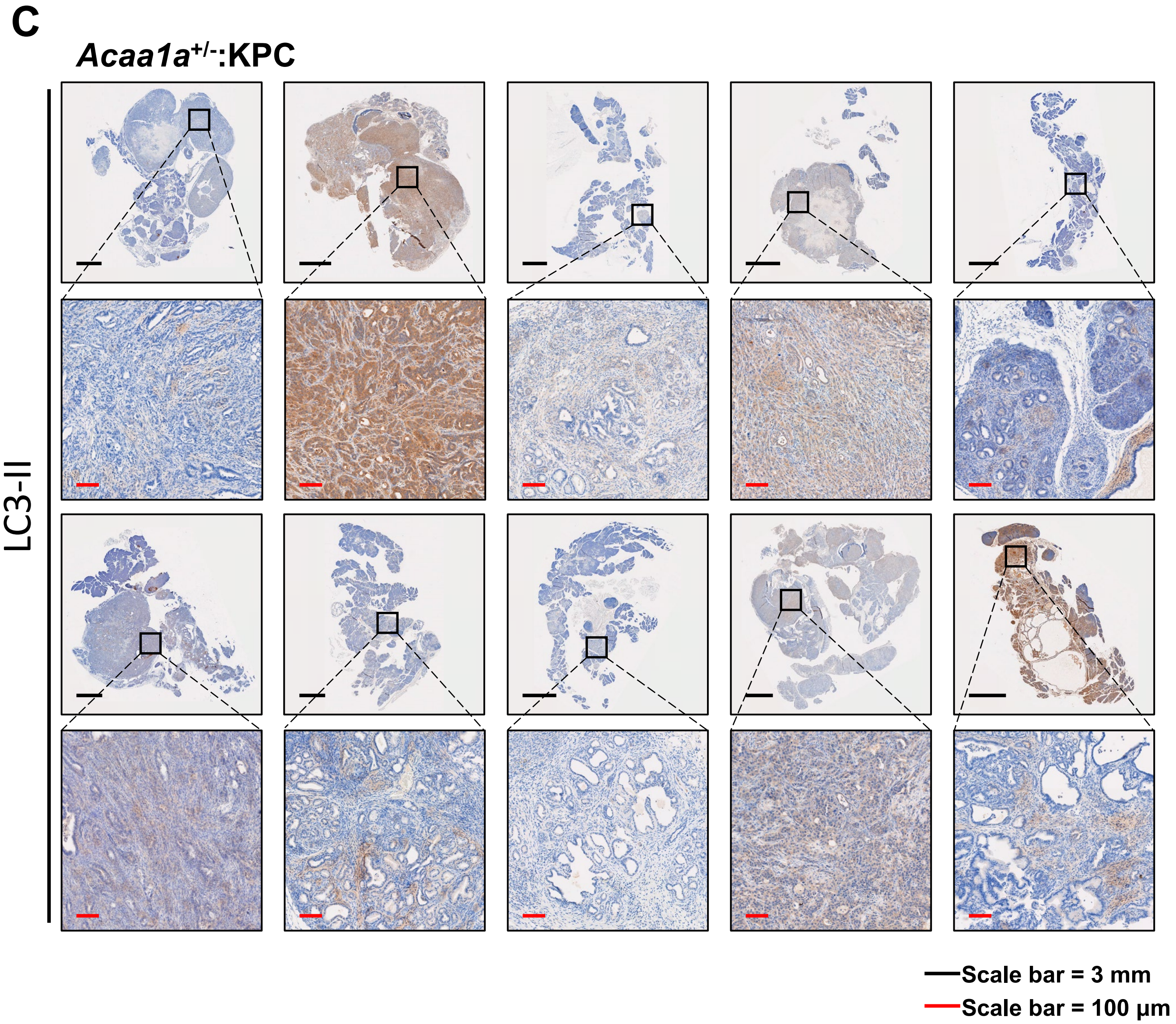

Supplementary Fig. 4

Representative images of H&E staining (A), immunohistochemical staining for KI-67 (1:1000) (B), and LC3 II (1:750) (C) in pancreatic tissues from KPC and *Acaa1a*$^{+/-}$;KPC mice. Images correspond to Fig. 6D. Ki-67 and LC3-II staining were used to assess proliferation and autophagy, respectively. (n = 10, black scale bar = 3 mm, red scale bar = 100μm).
